# Salvage surgery of the hip in non-ambulatory cerebral palsy: a systematic review

**DOI:** 10.1530/EOR-2025-0211

**Published:** 2026-07-01

**Authors:** Naomi Khan, Tara Korbal, Daniel Gould, Erich Rutz

**Affiliations:** ^1^ Department of Paediatrics, Bob Dickens Orthopaedic Center for Education and Research, University of Melbourne, Parkville, Victoria, Australia; ^2^ Department of Orthopaedics, The Royal Children's Hospital, Melbourne, Victoria, Australia; ^3^ Murdoch Children's Research Institute, Melbourne, Victoria, Australia; ^4^ Department of Paediatrics, Bob Dickens Chairs, Paediatric Orthopaedic Surgery, The University of Melbourne, Melbourne, Victoria, Australia; ^5^ Medical Faculty, University of Basel, Basel, Switzerland; ^6^ School of Health and Biomedical Sciences, Royal Melbourne Institute of Technology (RMIT University), Melbourne, Victoria, Australia

**Keywords:** cerebral palsy, salvage surgery, hip dislocation, proximal femoral resection, hip arthrodesis, hip arthroplasty, GMFCS, valgus osteotomy

## Abstract

**Purpose:**

- Individuals with severe cerebral palsy (CP) at Gross Motor Function Classification System (GMFCS) levels IV and V are at high risk of hip displacement causing pain, reduced sitting tolerance, and hygiene challenges. Salvage procedures such as proximal femoral resection, hip arthrodesis, or total hip arthroplasty are often required. This review evaluated preoperative characteristics, intraoperative variables, and postoperative outcomes of salvage hip surgery in non-ambulatory children and young adults with CP.

**Methods:**

- EMBASE (via Ovid), Medline and PubMed were searched from inception to October 1, 2024, following PRISMA guidelines and a registered PROSPERO protocol. Forward and backward citation chasing was undertaken prior to final submisson. Eligible English-language cohort studies and case series were included. Data were extracted in duplicate and quality was assessed using Methodological Index for Non-Randomized Studies (MINORS). A narrative synthesis was used due to the lack of consistent quantitative outcomes reported.

**Results:**

- Of 3,004 records screened, 42 studies (793 patients, 1,014 hips) were included. Excision was most common (*n* = 34 studies), followed by arthroplasty (*n* = 8) and arthrodesis (*n* = 4). Most patients were male (56%), and 81.6% were GMFCS level V. Excision procedures had the lowest operative times (mean 2.5 h) and blood loss (mean 232 mL). Preoperative indications included pain (93% of studies) and caretaker concerns including transfer ability, hygiene, and quality of life (83% of studies). Overall, perioperative complication rates were 13.9%. Heterotopic ossification occurred in 69% of studies, proximal femoral migration occurred in 52%, and 80% reported the need for secondary procedures.

**Conclusions:**

- Salvage surgery provides consistent pain relief and functional benefits in non-ambulatory CP but remains associated with frequent complications and repeat surgery.

## Introduction

Cerebral palsy (CP) is the most common physical disability of childhood impacting 1 in 700 children in Australia (1). The condition affects the development of movement and posture, causing activity limitations, commonly attributed to non-progressive disturbances that occurred in the developing fetal or neonatal brain (2). CP presents in several subtypes such as spastic, athetoid, or ataxic, with spastic CP being the most prevalent (3). Although individuals with CP are born with anatomically normal hips, chronic muscle spasticity, pathological weight bearing, and excessive femoral anteversion predispose to progressive hip subluxation and dislocation over time (3, 4). This is particularly common among non-ambulatory patients (Gross Motor Function Classification System (GMFCS) levels IV and V) as the risk of significant hip migration is nearly 90% (5). Clinically, this manifests as severe pain, impaired sitting, poor hygiene access due to adduction contractures, difficulty sleeping, and overall negative impact on quality of life for both the patients and caretakers (3, 4).

The goal of surveillance and preventative surgery is to avoid these consequences, but often attempts to preserve hip integrity through methods such as botulinum toxin injections, soft-tissue releases, and reconstructive osteotomies are not sufficient (3). In patients with CP, chronic muscle imbalance and abnormal joint loading can lead to progressive hip subluxation and eventual dislocation. Without timely intervention, this often results in painful, dislocated hips that necessitate salvage procedures such as proximal femoral resection arthroplasty (e.g. the Castle or McCarthy technique), valgus femoral osteotomy, hip arthrodesis, and total hip arthroplasty (3, 6, 7). Salvage hip procedures aim to restore function, relieve pain, and improve quality of life. Despite their widespread clinical use, substantial variability exists in indications, surgical techniques, perioperative complications, and functional outcomes across institutions. The literature is largely limited to retrospective case series with small sample sizes, non-standardized outcome measures, and heterogeneous follow-up periods, making it difficult to establish evidence-based guidelines for salvage interventions in CP-related hip disease. Moreover, complications that range from infection to persistent pain or loss of function are common but not uniformly reported.

To address this gap, we conducted a systematic review focusing on the outcomes of salvage hip surgeries in non-ambulatory children and young adults (<30 years) with CP (GMFCS levels IV and V). Specifically, we compared preoperative characteristics such as surgical indications and patient demographics, procedural approaches, intraoperative metrics (e.g. blood loss and complication rates), and postoperative functional outcomes (e.g. pain relief, sitting time, range of motion, and reoperation rates). By delineating indications, surgical approaches, and long-term results, we hope to inform clinical decision-making and identify areas requiring further prospective study.

## Methods

The review was conducted according to the Preferred Reporting Items for Systematic Reviews and Meta-Analyses (PRISMA) (8). The protocol was registered on March 22, 2025, and is available on PROSPERO (registration number: CRD420251008678). 

### Data sources and search strategy

The literature search strategies were developed and carried out by two of the authors (NK and TK) in collaboration with an experienced research librarian. The literature search was conducted on October 1, 2024. Three databases were searched: EMBASE (via Ovid), Medline and PubMed from inception to October 1, 2024, without date restrictions. Only English-language studies were included. The full search strategy (supplementary documents) combines terms for ‘cerebral palsy’, ‘salvage surgery’, ‘palliative hip surgery’, and procedure-specific keywords (e.g. ‘Girdlestone’, ‘McHale’, and ‘femoral head resection’) using both controlled vocabulary and free-text terms. In addition, forward and backward citation chasing of all included studies was undertaken prior to final write up and submission (9). This process did not yield additional studies beyond those captured through the database searches.

### Eligibility criteria

- Population: studies were included if greater than 50% of participants were non-ambulatory children and young adults (0–30 years) with CP (GMFCS levels IV and V) and chronic, painful hip subluxation/dislocation amenable to salvage surgery. This threshold was selected to ensure study populations were representative of the target group while allowing inclusion of studies where a small proportion of participants fell outside the strict criteria.
- Interventions: palliative and/or salvage hip procedures including proximal femoral resection (with or without interposition), subtrochanteric valgus osteotomy (± head resection), McHale/Shanz/‘Castle’ procedures, hip fusion (arthrodesis), and prosthetic arthroplasty (including total hip replacement).
- Study Designs: prospective or retrospective cohort studies, case series, and randomized controlled trials (RCTs) were eligible for inclusion.
- Exclusions: pelvic osteotomies (e.g. Dega and Pemberton), intertrochanteric osteotomies aimed at reconstruction, case reports, reviews, editorials, conference abstracts, and dissertations.

### Study selection and data extraction

Studies from all three databases were imported into the desktop reference manager Endnote 21 (10). This was followed by importing studies into Covidence (11) for deduplication. Studies were initially screened against inclusion criteria according to titles and abstracts independently by two reviewers (TK and NK). Full texts of potentially eligible studies were reviewed similarly. Conflicts throughout the screening process were resolved by consensus or a third, senior reviewer (ER). In a case series design, data regarding patients who fulfilled eligibility criteria were included and non-eligible data were disregarded.

Two authors (NK and TK) extracted data in duplicate using a standardized form, which was designed with input from all authors, including the senior author and pilot tested to ensure consistent data extraction. Extracted variables included study characteristics, patient demographics, indication for surgery, surgical details, intraoperative complications (Clavien–Dindo) (12), and postoperative outcomes. Where reported, data on prior hip or pelvic surgeries were extracted, including soft-tissue releases, femoral or pelvic osteotomies, and other earlier orthopedic interventions. Surgical laterality (unilateral/bilateral), staging (single- or two-stage), and concomitant procedures (e.g. adductor, psoas, or hamstring tenotomies) were also recorded. Non-surgical adjuncts for spasticity, such as intraoperative botulinum toxin injections, were documented when available. Investigators were not contacted for missing data.

### Risk of bias and quality appraisal

Risk of bias and quality appraisal was done by the first author (NK) and checked by the second author (TK). This was done using the Methodological Index for Non-Randomized Studies (MINORS), a validated scoring system that evaluates reporting quality and methodological soundness (13).

### Data synthesis

Due to heterogeneity in intervention and outcome, measured meta-analysis was not feasible, and instead, a narrative synthesis was conducted based on synthesis without meta-analysis reporting guidelines (14). The narrative synthesis focused on identifying trends in surgical indications, intraoperative techniques, complication profiles, and functional outcomes across various salvage procedures. Studies were grouped by surgical technique.

## Results

**Figure 1:**
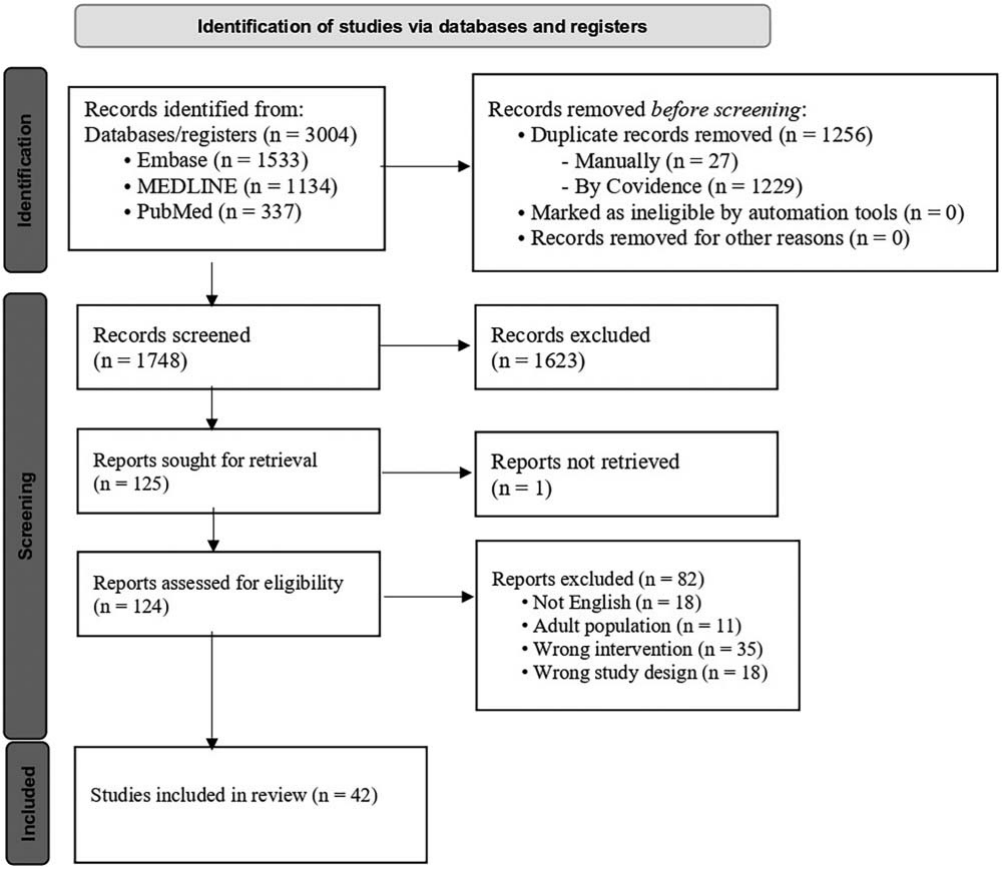
PRISMA flowchart of the selection process.

The initial search yielded 3,004 records. Of these studies, 42% (*n* = 1,256) were duplicates and removed. The remaining 1,748 studies were screened based on titles and abstracts, and a further 116 full-text articles were assessed for eligibility. A total of 42 studies fulfilled inclusion criteria and were included in the final synthesis. Covidence was used to help assess study eligibility. Figure 1 illustrates the PRISMA flow diagram.

### Risk of bias and quality assessment

**Figure 2:**
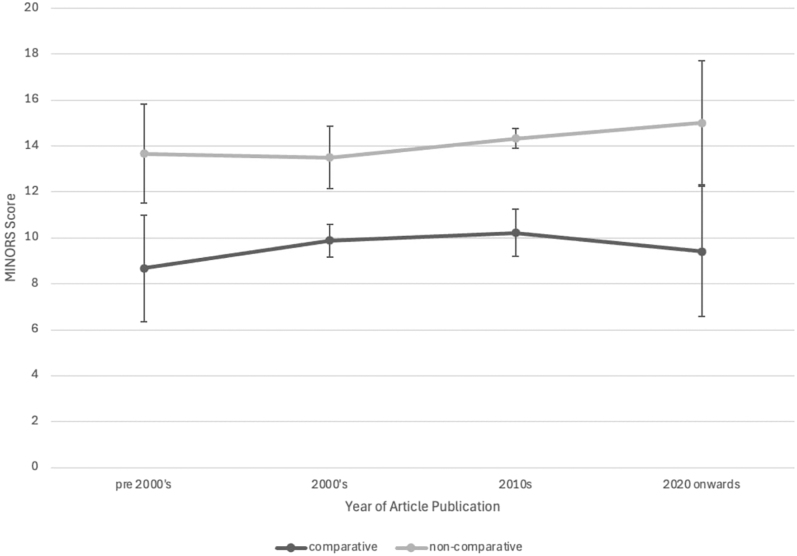
Mean MINORS score separated by time period of publication for comparative and non-comparative studies.

All included studies received a full scoring according to MINORS (13). The mean ± SD MINORS score for all non-comparative studies was 9.6 ± 0.9, and the mean ± SD MINORS score for all comparative studies was 13.3 ± 1.2. When MINORS was compared across decades of publication, there was no significant difference in the quality of studies (Fig. 2).

### Study characteristics

The included studies spanned from 1972 to 2024, consisting exclusively of retrospective case series and cohort designs (Table 1). No RCTs were identified. The majority were single-center investigations, and study sizes ranged from 2 to 63 patients. There were a total 947 patients and 1,227 hips that were operated on (excluding potential overlaps in cohort studies). Studies were published in various journals, with the most common being the *Journal of Pediatric Orthopedics* (*n* = 13 studies) (Table 1).

**Table 1:** Study demographics. Summary of included studies reporting salvage hip procedures in non-ambulatory children and young adults with cerebral palsy. Data presented include study design, journal impact factor, and surgical technique used. Values are presented as number of studies (and proportion of total number of studies).

Study characteristics	Values, *n* (%)
Decade of publication	
1971–1980	2 (5%)
1981–1990	4 (10%)
1991–2000	3 (7%)
2001–2010	11 (26%)
2011–2020	16 (38%)
2021 onwards	6 (14%)
Journal of publication	
*Journal of Pediatric Orthopedics*	19 (46%)
*The Bone & Joint Journal*	3 (7%)
*The Journal of Bone & Joint Surgery*	3 (7%)
*Developmental Medicine & Child Neurology*	2 (6%)
*Journal of Children’s Orthopedics*	2 (6%)
*Orthopedics & Traumatology: Surgery & Research*	2 (6%)
*Acta Orthopedia*	1 (2%)
*Acta Orthopedia Belgica*	1 (2%)
*Children*	1 (2%)
*Frontiers in Neurology*	1 (2%)
*International Orthopedics*	1 (2%)
*International Orthopedics – SICOT*	1 (2%)
*Journal of Pediatric Rehabilitation Medicine*	1 (2%)
*Orthopedics*	1 (2%)
*Orthopedic Clinics of North America*	1 (2%)
*Techniques in Orthopedics*	1 (2%)
*The Surgeon*	1 (2%)
Level of evidence	
Level 4	31 (74%)
Level 3	11 (26%)
Salvage surgery used*	
Excision	34 (81%)
Arthroplasty	8 (19%)
Arthrodesis	4 (10%)
Number of patients^†^	
<10	8 (19%)
11–20	17 (40%)
21–30	5 (12%)
>30	12 (29%)
Sex^‡^	
Majority male	17 (40%)
Majority female	14 (33%)
Equal number of male/female	2 (5%)
Not reported	9 (22%)
Mean age at time of surgery (years)^§^	
>10 to ≤15	20 (48%)
>15 to ≤20	16 (38%)
>20	7 (17%)
Age not specified	1 (2%)
Mean time of follow-up (years)^§^	
<5	25 (60%)
≥5	17 (41%)
Not reported	3 (7%)
GMFCS level^[^^§^^]^	
V	35 (83%)
IV	14 (33%)
III	5 (12%)
Not reported	6 (14%)

* Full name of specific procedure outlined in Appendix Table 1A. Some studies may use more than one procedure; thus, proportions may not add up; a full cohort break down is available in Appendix Table 1A.

^†^ Number of hips is mentioned in Appendix Table 1A.

^‡^ Sex is based on number of studies with more males vs females. Proportions may not add up as some studies use more than one procedure. A full break down is available in Appendix Table 1A.

^§^ Some studies include more than one patient cohort; thus, proportions may not add up; a cohort breakdown is available in Appendix Table 1A.

### Patient demographics

Table 1 depicts a summary of study characteristics. For the full details of each individual study, see Appendix 1, Table 1A (see section on Supplementary materials given at the end of the article). Functional status was most commonly classified using the GMFCS (15), with a predominance of individuals at levels IV and V (Table 1) (15). Specifically, 35 studies (83%) included patients classified as GMFCS level V (15, 16, 17, 18, 19, 20, 21, 22, 23, 24, 25, 26, 27, 28, 29, 30, 31, 32, 33, 34, 35, 36, 37, 38, 39, 40, 41, 42, 43, 44, 45, 46, 47, 48, 49, 50). Fourteen studies (33%) included patients at GMFCS level IV (6, 15, 18, 19, 21, 22, 23, 24, 28, 32, 33, 35, 37, 38, 46). Six studies (14%) did not report GMFCS classification (51, 52, 53, 54, 55, 56).

The sex distribution across studies was approximately 56.0% male and 44.0% female. The reported mean ages of participants ranged from 11.3 to 26.7 years, with a cumulative mean age of 16.4 years (Table 1).

### Preoperative indications

**Figure 3:**
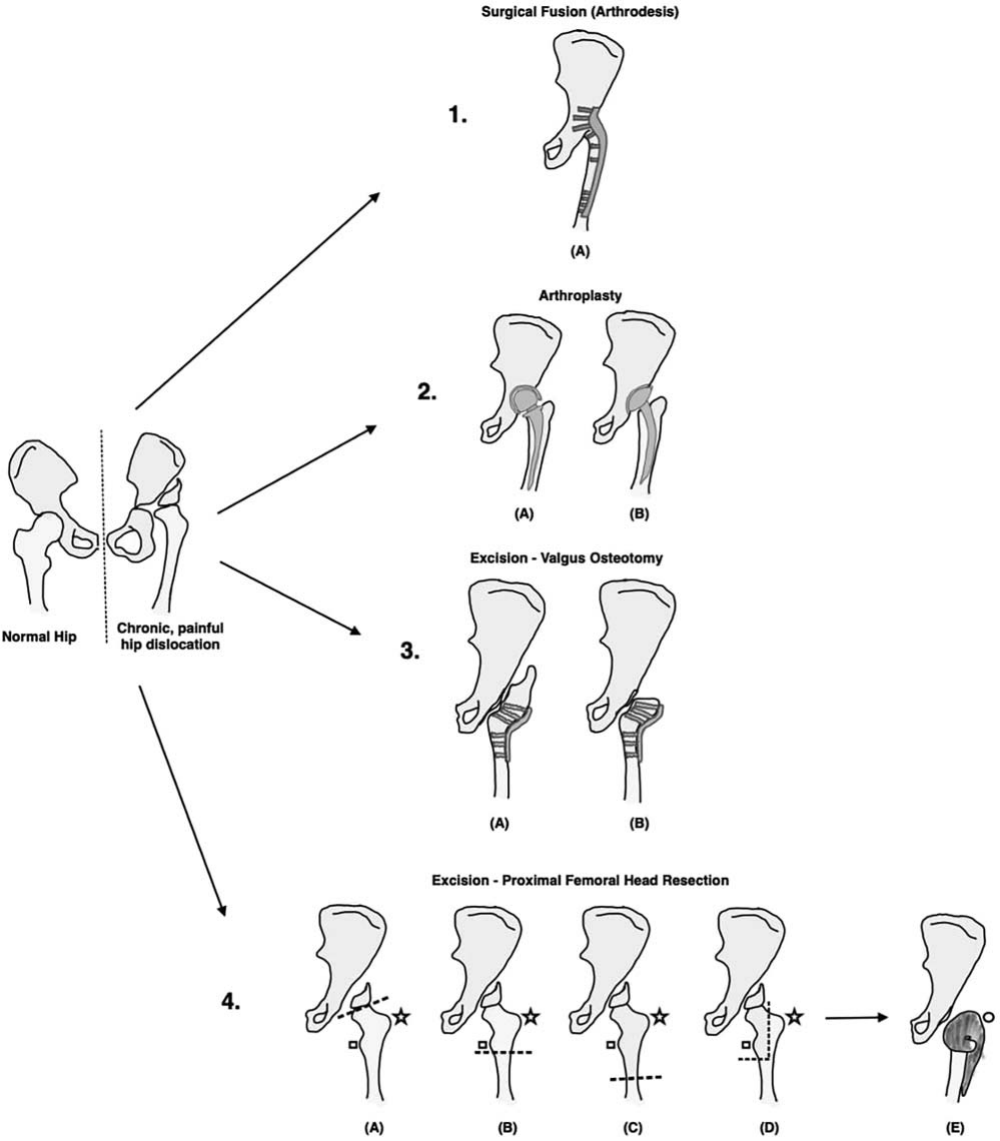
Schematic of salvage surgery techniques. 1) Arthrodesis/surgical fusion of the hip (6). 2A) Total hip arthroplasty (6). 2B) Shoulder prosthetic interpositional arthroplasty (31). 3A) Shanz subtrochanteric valgus osteotomy (57). 3B) McHale subtrochanteric valgus osteotomy with femoral head resection (43). 4) The star symbol represents greater trochanter. The square symbol represents lesser trochanter. 4A) Girdlestone – resection at the neck of femur (58). 4B) Castle – resection at the level of lesser trochanter (21). 4C) McCarthy – resection minimum 3 cm below the lesser trochanter (59). 4D) Schoenecker & Bauer – greater trochanter sparing resection (19). 4E) Surgical outcome of proximal femoral resection with vastus lateralis interposition represented by a circle symbol.

Studies were grouped by the surgery type as excision procedures – 34 studies (16, 17, 18, 19, 20, 21, 22, 23, 24, 29, 30, 32, 33, 34, 35, 36, 37, 38, 39, 40, 41, 42, 43, 45, 46, 47, 49, 50, 51, 52, 53, 54, 55, 56), arthroplasty – eight studies (28, 31, 38, 39, 44, 48, 49, 56), and arthrodesis – four studies (6, 25, 26, 27). An overview of the surgical approaches used in salvage surgery by group is presented in Fig. 3.

Across the included studies, 93% (*n* = 39/42) explicitly mentioned pain (6, 16, 17, 18, 19, 20, 21, 22, 23, 25, 26, 27, 28, 29, 30, 31, 32, 33, 34, 35, 36, 37, 38, 39, 40, 41, 42, 43, 44, 45, 46, 47, 48, 49, 50, 51, 52, 53, 54, 55, 56) as the primary indication for salvage hip surgery. Pain was often reported narratively, though a minority of studies employed standardized measures, such as visual analog scale (VAS), Likert-type assessments, or documentation of analgesic usage (17, 18, 41, 46, 49, 54). Some studies categorized pain using scales such as mild, moderate, or severe, with severe pain most frequently reported (23, 25, 26, 37). Notably, 24 out of 42 screened studies (57%) explicitly quantified that 100% of the cohort experienced pain (18, 19, 20, 22, 23, 25, 27, 28, 29, 31, 32, 33, 34, 38, 43, 47, 48, 49, 50, 51, 52, 53, 55, 56).

Additional indications included chronic hip dislocation or subluxation, as well as functional impairments such as difficulties with seating, perineal care, hygiene, and transferability. Although sitting tolerance was inconsistently quantified, a minority of studies provided specific data. Among those, most patients had a sitting time of less than 60 min, with the longest reported time being 240 min and the shortest under 30 min (6, 17, 18, 31, 34, 39, 46, 49, 53, 54, 55). Notably, 14 patients across various studies were reported to be completely unable to sit preoperatively (17, 21, 40, 47, 49, 53).

Several studies reported prior hip procedures earlier in the disease course although the timing of these interventions was not specified. These most commonly included soft-tissue releases and reconstructive procedures such as femoral or pelvic osteotomies performed in attempts to maintain hip stability (6, 16, 17, 19, 22, 23, 25, 26, 27, 31, 33, 36, 38, 38, 41, 43, 44, 46, 47, 48, 50, 51, 55, 56). Patients undergoing excisional salvage procedures frequently had a history of previous reconstructive osteotomies or soft-tissue procedures that had failed to prevent progressive hip displacement and pain (6, 17, 18, 19, 20, 22, 23, 33, 41, 43, 46, 47, 50, 51, 52, 55, 56). In contrast, arthroplasty procedures were more commonly performed in patients with persist pain or function limitations following previous excisional salvage procedures prompting consideration of prosthetic reconstruction (6, 44, 48, 56).

Reporting of surgical laterality varied across the included studies with several studies describing both unilateral and bilateral procedures. In excisional salvage procedures, the staging of bilateral procedures was dependent on anesthetic fitness; patients deemed fit underwent single-stage procedures, while those unfit for prolonged surgical times underwent two-staged procedures (18, 45, 50). In comparison, arthroplasty procedures that required bilateral interventions underwent a two-stage surgical plan with an average of 14 months in between procedures (44). This was due to poor clinical outcomes of one-staged bilateral arthroplasty in the fragile CP patient population, citing that performing one hip at a time with sufficient time for rehabilitation allowed CP patients to rely on the contralateral hip for stability while they recovered, reducing the incidence of repeat hip dislocations (44). All patients who underwent arthrodesis had unilateral procedures (6, 25, 26, 27).

### Caretaker concerns

Caretaker concerns were reported in 35 of the 42 included studies (83%). Among all studies, hygiene-related issues were cited in 32 (76%), including difficulties with perineal care, restricted access to the perineal region, toileting challenges, and increased demands on nursing care (6, 16, 17, 18, 19, 20, 21, 22, 23, 28, 29, 30, 31, 32, 33, 34, 36, 37, 38, 40, 42, 43, 44, 45, 46, 48, 49, 50, 51, 54, 55, 56). Transfer difficulties were reported in 11 studies (26%) (6, 18, 28, 36, 40, 42, 46, 49, 50, 53, 55). Quality of life concerns were identified in seven studies (17%) (36, 41, 43, 44, 48, 54, 56).

### Radiological measurements

Radiographic data were reported in 24 studies (57%) (6, 16, 18, 19, 23, 26, 27, 30, 31, 33, 34, 37, 39, 40, 41, 42, 44, 45, 46, 47, 48, 49, 50, 55). Common findings included deformed femoral heads, shallow dysplastic acetabula, and moderate-to-severe hip subluxation or dislocation. Importantly, all studies that reported radiographic data cited the presence of hip displacement (6, 16, 18, 19, 23, 26, 27, 30, 31, 33, 34, 37, 39, 40, 41, 42, 44, 45, 46, 47, 48, 49, 50, 55).

Quantitative assessments of hip migration were included in a subset of studies. Two studies specifically referenced the Reimers migration percentage (23, 33). One study reported that all patients had migration percentages >33%, with 12 patients exceeding 100%, indicating complete dislocation (33). Another study utilized the Melbourne Cerebral Palsy Hip Classification System (MCPHCS) (50, 60). The study reported a mean femoral head migration of 73%, with an average Grade 5 deformity on the MCPHCS (50, 60). Femoral head deformity was not evaluated using the Rutz femoral head deformity classification (61).

### Presence of scoliosis

Nine studies (21%) reported the presence of scoliosis in their patient populations (17, 20, 25, 31, 43, 44, 46, 47, 50). Scoliosis is a common comorbidity in children with severe CP, particularly in non-ambulatory individuals classified as GMFCS levels IV and V (50). No studies quantified the severity of spinal deformity. Spinal fusions were reported to be performed both before and after salvage surgery but the rationale behind the decision was not reported in most studies (17, 20, 25, 31, 43, 44, 46, 47, 50). One study explained that if hip pain is not the main concern and there is some degree of flexion of the spine, scoliosis should be corrected prior to the undertaking of salvage surgery (44). Three studies described the development of new scoliosis after the procedure, citing this as a reason for caretaker dissatisfaction (26, 51, 53), and one study described that there was no effect on already present scoliosis secondary to salvage procedures (47).

### Perioperative outcome

Table 2 summarizes intraoperative metrics; for the full characteristic of each study, see Appendix 1, Table 1B. Blood loss was reported in 11 studies, with excision procedures generally associated with lower weighted average intraoperative blood loss (232.5 mL) compared with arthroplasty (527.3 mL) and arthrodesis (1,450 mL). Operative time ranged widely across procedures (0.9–3.7 h) (6, 18, 20, 22, 28, 31, 32, 33, 41, 44, 54). Perioperative complications were inconsistently reported, with the most commonly described being superficial wound infection (17, 23, 25, 26, 27, 30, 33, 41, 42, 46, 49, 53, 54). Only one study applied complication grading (e.g. Clavien–Dindo) (35). Concomitant procedures were variably reported across studies. Several studies described additional soft-tissue procedures performed at the time of salvage surgery (6, 17, 18, 20, 26, 26, 31, 38, 42, 43, 44, 46, 47, 51, 53). Adductor, psoas, and hamstring tenotomies were most commonly reported and were preformed to allow neutral hip positioning in those with severe contractures and to reduce spastic muscle imbalance (6, 42, 44, 47, 53). Non-surgical strategies were also used to address spasticity, such as intraoperative botulinum toxin injections in previously mentioned muscles as an alternative to additional tenotomies (18, 46).

**Table 2:** Summary of perioperative outcomes for proportion of studies reporting length of surgery, blood loss, and immediate intraoperative complications.

Outcomes	Surgery type		
	Excision	Arthroplasty	Arthrodesis
Total studies, *n*	34	8	4
Length of surgery, minutes (mean)*			
Not reported	27 (79%)	5 (61%)	3 (75%)
<90	0 (0%)	1 (13%)	0 (0%)
≥90 to <150	5 (15%)	0 (0%)	0 (0%)
≥150 to <200	1 (3%)	1 (13%)	0 (0%)
≥200	1 (3%)	1 (13%)	1 (25%)
Blood loss in mL (mean)*			
Not reported	27 (79%)	5 (63%)	3 (75%)
<500	7 (21%)	2 (25%)	0 (0%)
≥500 to <1,000	0 (0%)	1 (12%)	0 (0%)
≥1,000	0 (0%)	0 (0%)	1 (25%)
Intraoperative complications^†^			
Skin complications	45	0	3
Infection	3	5	0
Fractures	2	3	5
Transfusion	10	18	1
ICU admission	4	2	0
Other	15	6	5
Length of postoperative stay in days (mean)*			
Not reported	23 (67%)	7 (88%)	3 (75%)
<10	8 (24%)	1 (12%)	0 (0%)
≥10	3 (9%)	0 (0%)	1 (25%)

* Recorded as a proportion out of the total number of articles in the specified surgical category.

^†^ Counted as the number of patients that experienced the intraoperative complication(s). Specific complications listed in Appendix Table 1B.

### Complications

Complications were reported using various criteria across studies; however, for consistency, we categorized complications according to the Clavien–Dindo–Sink classification system (12). Across all included studies, a total of 951 patients underwent salvage procedures, with an overall complication rate of 13.9% (Table 2). Excision was the most commonly performed procedure (*n* = 779), with 114 reported complications (14.6%) (9, 10, 11, 12, 13, 14, 15, 16, 17, 22, 23, 25, 26, 27, 28, 29, 30, 31, 32, 33, 34, 35, 36, 38, 39, 40, 42, 43, 45, 46, 47, 48, 49, 50). Arthrodesis was performed in 62 patients, with 12 complications (19.4%) (18, 19, 20, 44), while arthroplasty was performed in 110 patients and had the highest complication rate, with 32 complications (29%) (21, 24, 31, 32, 37, 41, 44, 50).

**Figure 4:**
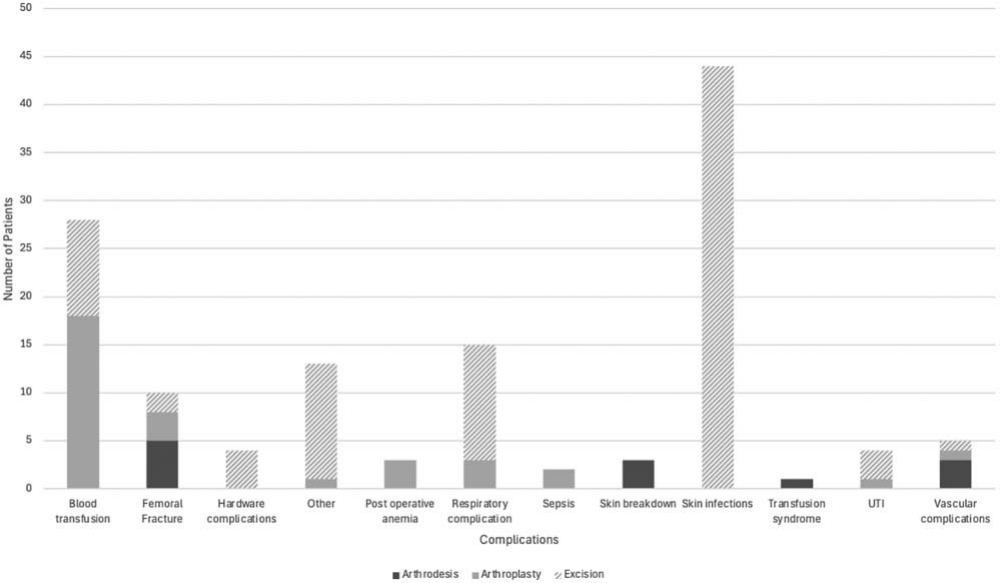
Complications following salvage procedures (arthrodesis, arthroplasty, and excision).

When classified using the Clavien–Dindo–Sink system, most complications were Grade I or II, including superficial wound infections (*n* = 15, all following excision) (10, 12, 16, 23, 26, 35, 39, 47, 48), and skin breakdown (*n* = 18; 15 after excision (10, 13, 34, 35, 42) and 3 after arthrodesis (18, 19, 20)). More severe complications (Grade III or higher) included fractures (*n* = 10; 5 following arthrodesis (18, 19, 20), 3 after arthroplasty (21, 24), and 2 after excision (10, 35)) and significant blood loss or transfusion (*n* = 31; 21 in arthroplasty (37, 41) and 10 in excision (9, 12, 15)). Respiratory complications were classified as Grade IVa, as they often required ICU admission without resulting in permanent disability. Neither Grade IVb complications nor Grade V complications were reported in any of the included studies. A comprehensive overview of all reported complications and their classifications is provided in Appendix 1, Table 1D, and Fig. 4.

### Postoperative outcomes

The follow-up duration varied considerably (1–14.5 years), with a mean follow-up of 4.8 years across studies. Table 3 summarizes postoperative metrics. For the full details of each included individual study, refer to Appendix 1, Table 1C and 1D. Pain, functional outcomes including sitting tolerance, caregiver-reported measures, and follow-up duration were the most consistently reported variables and therefore formed the primary focus of analysis. Given that, the principle aims of salvage procedures are pain relief and improved caregiving. Accordingly, a stratified summary of pre- and postoperative pain, functional outcomes, caregiver-reported measures, and follow-up duration is provided in Appendix 1, Table 1D, to facilitate comparison across studies.

**Table 3:** Summary of postoperative functional outcomes. Outcomes include pain relief, sitting tolerance, ambulation potential, and range of motion, stratified by surgical techniques.

Functional outcomes	Surgery type		
	Excision	Arthroplasty	Arthrodesis
Total studies, *n*	34	8	4
Pain relief*			
100%	17 (50%)	3 (38%)	3 (75%)
75–99%	12 (35%)	4 (50%)	1 (25%)
50–74%	4 (12%)	1 (12%)	0 (0%)
25–49%	0 (0%)	0 (0%)	0 (0%)
Not reported	1 (3%)	0 (0%)	0 (0%)
Persistent pain	7 (21%)	3 (38%)	0 (0%)
Sitting time^†^			
Improved by 30–60 min	0	0	0
Improved by > 60 min	11	2	0
Improved but not quantified by time	13	4	4
Not reported	10	2	0
Not improved or worsened	4	0	0
Ambulation^‡^			
Improved	1	0	4
Worsened	0	0	1
Range of motion^§^			
Improved abduction	9	1	0
Improved adduction	2	0	0
Improved flexion	9	2	0
Improved extension	6	1	0
Improved internal rotation	2	0	0
Improved external rotation	2	0	0
Improved total range of movement	11	4	1
No improvement	1	0	0
Worsened	2	2	0
Not reported	14 (41%)	2 (25%)	3 (75%)

* Pain relief reported by articles in percentage proportion of patient cohort. Persistent pain recorded as articles citing any percentage of patient cohort experiencing persistent pain post-procedure, recorded as a proportion out of the total articles in that surgical category. Further details are given in Appendix Table 1C.

^†^ Articles citing any percentage of the cohort experiencing no improvement in sitting or worsened were recorded. Further details are given in Appendix Table 1C.

^‡^ Most patient cohorts remained non-ambulatory. Articles citing worsening and improvement of ambulation in patients were recorded. Articles may overlap. Further details are given in Appendix Table 1C.

^§^ Articles citing range of movement changes with different parameters were recorded. Articles that mentioned more than one parameter change were accounted for; thus, articles may overlap. Articles that did not report range of movement parameters are listed as a proportion of total articles. Further details are given in Appendix Table 1C.

### Pain relief

Pain relief was one of the most consistently reported following salvage hip procedures. Majority of patients experienced reductions in pain, regardless of the procedure type (Table 3). While standardized pain scores (e.g. VAS (18, 46), numeric rating scale (NRS) (38, 41, 54), or validated surveys such as CPCHILD™ (36, 42) or PedsQL™ (36)) were reported in a minority of studies, narrative and caretaker-reported outcomes consistently indicated subjective improvement.

In total, 20 studies reported complete or near-complete pain relief in all patients (6, 16, 17, 18, 19, 20, 21, 23, 25, 26, 27, 28, 29, 30, 31, 36, 43, 44, 47). In studies using quantitative measures, significant improvements in pain scores were consistently observed. For example, Abu-Rajab *et al.* reported a drop in mean VAS from 8.4 preoperatively to 3.3 postoperatively (*P* < 0.0001) (16), while Duporte *et al.* noted a reduction from 5.4 to 2.1 on the NRS in patients undergoing hip reconstruction (*P* < 0.001) (29). Other studies reported reductions in pain across multiple domains, sitting, lying, and perineal care, with statistical significance (*P* < 0.05 in most) (18, 24, 36, 38, 42, 44, 46, 54).

### Sitting tolerance

Sitting tolerance improved in most patients following salvage surgery, though reporting methods varied widely across studies (Appendix 1, Table 1C).

In excision procedures, improvements were observed in all patients in several series (16, 17, 18, 19, 20, 21, 29, 30, 32, 34, 38, 39, 41, 42, 43, 45, 46, 47, 49, 50, 53, 55). One study reported six non-sitters who all gained sitting ability postoperatively (27). Sitting time increased from 30 to 120 min in one case series (18), while another documented an average gain of 3 h (41). Multiple studies reported that all patients could sit for 3–4 h or longer after surgery (17, 34, 39, 41, 43, 45, 49).

Arthroplasty outcomes were also positive but described in fewer studies. One series reported that all 12 patients with seating difficulties experienced improvement postoperatively (38). In another, THA patients sat up to 10 h (female) and 5 h (male) (39).

Arthrodesis was also associated with improved sitting in majority of reported patients. In one study, six bedridden patients improved to sitters (27), while in another study, five out of seven patients improved from bedridden to sitters (25). Studies did not quantify the amount of time patients were able to sit pre- or post-procedure.

### Caretaker-reported outcomes

Caretaker perspectives were most commonly reported in studies evaluating excision surgeries, with 27 studies documenting caretaker feedback (Appendix 1, Table 1D) (16, 17, 18, 19, 20, 21, 23, 29, 30, 32, 33, 34, 36, 37, 38, 40, 41, 42, 45, 46, 49, 50, 51, 53, 54, 55, 56). Several studies captured overall satisfaction ratings, with caretakers describing postoperative outcomes as ‘much better’ or ‘a bit better’ in terms of comfort and care logistics as well as ‘satisfied’ or ‘very satisfied’ with the overall results of the surgery (29, 33, 37, 41, 46, 51, 54). Three studies used CPCHILD™ (62). All studies showed improved scores on this questionnaire, indicating better quality of life post-excisional salvage intervention (36, 42, 50).

Arthrodesis procedures were associated with fewer caretaker-reported outcomes, with only two studies mentioning such data (Appendix 1, Table 1D) (26, 27). Patient satisfaction was reported for all cases.

All arthroplasty procedures reported at least >50% of caretakers would recommend this procedure, while one study reported that all caretakers were happy with final results and recommend the surgery to be performed on others (Appendix 1, Table 1D) (28, 31, 38, 56).

### Range of motion

Range of motion (ROM) improvements were reported in studies evaluating excision procedures, with 19 studies describing functional gains (Table 3) (17, 18, 19, 20, 21, 34, 36, 37, 39, 40, 42, 43, 45, 46, 47, 49, 51, 55, 56). Studies frequently documented increases in flexion and abduction, and some quantified gains explicitly; for example, one study noted a mean total hip ROM of 118.3° ± 30.5°, while others described ‘marked improvement’ or ‘conversion of adduction deformities to abduction postures’ (18, 20, 51).

In contrast, only one arthrodesis study reported ROM data following arthrodesis, which stated there were improvements but were not quantified (Table 3) (6).

Six out of eight studies utilizing arthroplasty as their surgical technique reported outcomes for ROM (Table 3). Some studies reported outcomes as excellent, good, fair, or poor, and in these studies, >50% of patients reported excellent or good outcomes with ROM (28, 48, 56). One study quantified ROM improvements, which stated flexion >80 degrees showed significant improvement (*P* > 0.05) (44). Other studies did not quantify values but stated all patients showed improvements in ROM (6, 39).

### Heterotopic ossification

Heterotopic ossification (HO) was a frequently encountered complication, particularly in excision-based surgeries, with 26 studies reporting radiographic or clinical evidence of HO (Appendix 1, Table 1D) (16, 17, 19, 22, 23, 24, 29, 30, 32, 35, 36, 37, 38, 39, 40, 41, 43, 45, 46, 49, 50, 52, 53, 54, 55, 56). Five studies showed that 100% of their patients showed radiographic evidence of HO (17, 39, 40, 50, 52). Several studies used the Brooker classification (63). In all of these studies, majority of patients who had HO were rated as grade 1 or grade 2 (16, 17, 35, 36, 37, 45, 50, 54). In contrast, arthrodesis procedures had no reporting on HO (Appendix 1, Table 1D).

Six out of eight arthroplasty studies reported HO (Appendix 1, Table 1D) (6, 31, 38, 39, 48, 56). The results varied as one study determined no patients had observable incidence of HO in follow-up radiograph (28), while another study determined an average grading of 1.4 on the Brooker classification system (48). Four studies demonstrated >50% of the cohort showing radiographic evidence of HO (6, 31, 38, 39).

### Proximal femoral migration

Proximal femoral migration was primarily associated with excision procedures, with 21 studies documenting such findings (Appendix 1, Table 1D) (16, 17, 19, 20, 22, 30, 32, 35, 36, 37, 40, 41, 42, 43, 45, 46, 47, 49, 50, 54, 55). Some studies showed all patients demonstrated some degree of proximal femoral migration (17), while other studies showed insignificant amounts (20, 36, 43, 46). Some showed quantitative values for migration, for example cited a mean migration distance of 10.7 mm (54). Other studies quantified this by comparing migration to the levels around the acetabulum with more patients showing migration at the level of the acetabulum (16, 40, 49).

Studies involving arthrodesis did not explicitly report on femoral migration (Appendix 1, Table 1D).

Two out of eight studies using arthroplasty reported on femoral migration. Both studies exhibited some level of proximal femoral migration although quantitative values were not explicitly mentioned (Appendix 1, Table 1D) (6, 28).

### Reoperation rates and surgical failures

Mechanical complications and surgical revisions were most frequently reported in excision surgeries, appearing in 29 studies (Appendix 1, Table 1D) (16, 17, 18, 19, 20, 22, 23, 24, 30, 32, 33, 35, 36, 37, 38, 39, 40, 41, 42, 43, 46, 47, 49, 50, 51, 53, 54, 55, 56). These included persistent pain, infection, fractures, and most notably, hardware failure requiring revision. In one series, 31% of patients experience hardware failure that required revision (36).

In arthrodesis, mechanical complications were reported in four studies, primarily involving non-union or pseudarthrosis (Appendix 1, Table 1D) (6, 25, 26, 27).

All studies using arthroplasty reported surgical revisions and failures post-procedure (6, 28, 31, 38, 39, 44, 48, 56). The most common reasons for revision were persistent pain, hardware failure requiring removal of implants, and fractures (Appendix 1, Table 1D). Figure 1 of Appendix 1 outlines procedure-specific complications.

### Mortality

Mortality was explicitly reported in eight of the 42 included studies (19%) (Appendix 1, Table 1D) (17, 30, 33, 37, 38, 39, 41, 44). All deaths were noted as unrelated to the surgical procedure with variable time periods post-procedure (17, 33, 37, 38, 39, 41, 44).

## Discussion

This systematic review examines salvage hip procedures in non-ambulatory patients with CP, focusing on indications, perioperative course, and outcomes. Reporting remains heterogeneous, with limited use of validated measures and inconsistent follow-up, hindering pooled analysis and guideline development.

Prior reviews summarized salvage surgery in CP (3, 64, 65, 66) but did not stratify outcomes by the procedure type or synthesized complication profiles. This review is the first to organize findings by the procedure category, integrate perioperative data, and evaluate heterogeneity using standardized tools (PRISMA and MINORS), providing an updated overview and highlighting evidence gaps.

Consistent with prior reviews (3, 64, 65, 66), the main indications were intractable pain (>90% of studies), impaired sitting, hygiene difficulties, and progressive hip disease in GMFCS level IV and V patients. Pain was often qualitatively described with limited use of validated measures (e.g. CPCHILD™ or PedsQL™) reflecting assessment challenges in cognitively impaired patients. Radiographic findings also consistently showed advanced pathology, including severe femoral head migration and deformity. Scoliosis was frequently present which further complicates hip biomechanics, contributing to pelvic obliquity and worsening displacement, though its role in surgical planning remains unclear with limited guidance on timing of spinal fusion (17, 20, 25, 31, 43, 44, 46, 47, 50, 66). Morin *et al.* (44) suggested that if hip pain is not the main concern and flexion is present in the spine, then scoliosis should be managed first; however, nearly all patients have indications of unretractable pain as their main indication for surgery.

Findings suggest salvage procedures occur within a stepwise pathway, progressing from soft-tissue or reconstructive surgery to excisional procedures, with arthroplasty reserved for failed cases. Most patients had prior hip surgery, though its impact on outcomes is underreported (48).

The selection of salvage procedures varies and was rarely well described. Influencing factors included surgeon preference, severity of hip deformity, age, acetabular degeneration, and medical complexity. Excision was favored in medically fragile patients due to lower operative burden (21), whereas arthroplasty was considered in older patients with preserved bone stock or previously failed excisional techniques. Arthrodesis is now rarely used and was historically reserved for selected patients without spinal deformity as this increased the rates of poor surgical outcome (6, 44). The lack of standardized criteria limits clear indications.

It is important to emphasize that bony reconstruction should always be the first-line surgical approach in managing hip displacement in CP patients as it prioritizes preservation of anatomy and function (3). Salvage procedures should be reserved as secondary options when reconstruction is not feasible or has failed (3).

Perioperative reporting was inconsistent, with variability in operative time, blood loss, transfusion rates, and ICU utilization. Excision procedures generally involved shorter operative times with less blood loss, while arthroplasty and arthrodesis carried higher complication rates. Only one study used a standardized framework (Clavien–Dindo–Sink) to grade complications, limiting comparability (35). Unlike other reviews, which did not classify perioperative complications using this scale (3, 64, 65, 66), this approach offers greater standardization in reporting outcomes such as disability, ICU admission, permanent deficits, mortality, prolonged hospital stays, and less acute complications (12). Wider adoption would improve benchmarking of morbidity.

Postoperative pain relief was the most consistent and clinically meaningful postoperative outcome, with nearly all studies reporting significant reductions in patient discomfort, which was consistent with other reviews (3, 64, 65, 66). Postoperative pain relief was reflected in longer sitting tolerance, improved range of motion, and enhanced overall quality of life, enabling patients to carry out daily activities with little to no discomfort. However, substantial heterogeneity existed in how these outcomes were reported and measured across studies. To address this, a structured summary of pain relief, functional outcomes, and follow-up duration stratified by surgical technique is provided in Appendix 1, Table 1E, to facilitate comparison across studies.

Key findings include higher migration and revision rates associated with femoral head excision, greater blood loss and intraoperative morbidity in arthroplasty, and infrequent but severe complications associated with arthrodesis, which is seldom used today. The high complication rates reported in arthrodesis and arthroplasty compared with excision procedures are consistent with other reviews (3, 64). However, the small sample size of studies including these procedures should be noted as this may introduce bias into the results.

An addition to this evolving surgical landscape is the Bauer and Schoenecker osteotomy. Modifying the Castle-type proximal femoral resection by securing a retained greater trochanter with its musculature to the capsular arthroplasty and femoral shaft would compartmentalize the femur, thereby decreasing proximal femoral migration and HO. In our review, HO (69%) and proximal femoral migration (52%) were common complications, and the Bauer and Schoenecker technique (19), designed to reduce these issues, represents a key advancement toward improved long-term hip stability and patient comfort. However, thus far, to the best of our knowledge, there are no reported long-term studies.

Future studies should prioritize prospective, multicenter data collection with standardized outcome measures. While randomized trials are unlikely to be feasible, given the impossibility of blinding, the high-risk nature of the population, and the importance of caretaker preference, prospective cohort designs could improve data quality. Routine adoption of validated tools such as CPCHILD™ (62), PedsQL™ (67), and standardized pain scales would facilitate longitudinal tracking of meaningful outcomes.

Strengths of this review include a prospectively registered protocol (PROSPERO) (68), adherence to PRISMA (8) frameworks, and rigorous methodology including dual reviewer screening, duplicate data extraction, and risk of bias assessment using MINORS (13) criteria.

However, our findings are limited by several factors. Only English-language, full-text articles were included, resulting in the exclusion of 18 non-English studies and potential selection bias. Heterogeneity in design, outcomes, and follow-up precluded meta-analysis and reduced the strength of our conclusions. Many studies lacked reporting of key surgical variables, including concomitant procedures, previous surgical history, and surgical laterality. These factors may influence outcomes following salvage surgery but were insufficiently described in many studies.

## Conclusion

Salvage hip surgery in non-ambulatory CP patients (GMFCS levels IV and V) provides consistent pain relief and improved functions, particularly for sitting tolerance and hygiene. However, evidence is limited by retrospective designs, heterogeneous surgical techniques, and inconsistent reporting, making it difficult to identify a clearly superior approach. Future research should focus on prospective, multicenter studies with standardized outcome measures. Reconstructive surgery remains the preferred approach where feasible, with salvage procedures reserved for symptomatic, non-reconstructable hips.

## Supplementary materials



## ICMJE Statement of Interest

The authors declare that there is no conflict of interest that could be perceived as prejudicing the impartiality of the work reported.

## Funding Statement

TK, DG, and ER are supported by the Lorenzo and Pamela Galli Medical Research Trust.
