# Supplementary material for: Salvage surgery of the hip in non-ambulatory cerebral palsy: a systematic review
Source: EFORT Open Rev. 2026 Jul 1;11(7):778–90. doi: 10.1530/EOR-2025-0211 (PMC13326878; doi:10.1530/EOR-2025-0211)
Supplement: Supplementary file 1 [file supplementary_materials.pdf]

Appendix 1 Table 1A. Study Demographics

Summary of included studies reporting salvage hip procedures in non-ambulatory children and young adults with cerebral palsy. Data presented include: study design, journal impact factor, and surgical technique used.

First Author (Year)	Journal (Impact Factor)	Level of Evidence	Salvage Surgery Used	Patients (Hips)	% Male	Mean Age at Time of Surgery (y)	Mean Time to Follow up (y)	GMFCS Score
Hoffer (1972)	Developmental Medicine & Child Neurology (3.8)	Level 4 - Retrospective Case series	Femoral head and neck resection	2 patients (4 hips)	0%	15	N/A	V (2)
Castle (1978)	The Journal of Bone & Joint Surgery (4.7)	Level 4 - Retrospective Case series	Castle	12 patients (14 hips)	N/A	12	2	IV, V
Koffman (1981)	Orthopedic Clinics of North America (1.4)	Level 3 - Retrospective Cohort study	Castle; Total Hip Arthroplasty	6 patients (10 hips); 2 patients (3 hips)	N/A; 33%	19; 24	8; N/A	V (6); V (2)
Baxter (1986)	Journal of Pediatric Orthopedics (1.4)	Level 4 - Retrospective Case series	Castle	4 patients (5 hips)	50%	14.5	N/A	V (4)
Root (1986)	The Journal of Bone & Joint Surgery (4.7)	Level 3 - Retrospective Cohort study	Arthrodesis; Total Hip Arthroplasty	8 patients (8 hips); 10 patients (10 hips)	50%; 44%	22; 24.4	6.75; 6.75	III (6), IV (2); III (7), IV (3)
McHale (1990)	Journal of Pediatric Orthopedics (1.4)	Level 4 - Retrospective Case series	McHale	5 patients (6 hips)	N/A	Young adolescents and one adult age 30	3.1	V (5)
Barrie (1996)	Journal of Pediatric Orthopedics B (0.9)	Level 4 - Retrospective Case series	Proximal femoral resection	6 patients (7 hips)	N/A	15.6	1.4	N/A
Gabos (1999)	Journal of Pediatric Orthopedics B (0.9)	Level 3 - Retrospective Cohort study	Proximal Femoral Arthroplasty with shoulder prosthesis	11 patients (14 hips)	55%	17	5	V (11)
Widmann (1999)	Journal of Pediatric Orthopedics (1.4)	Level 4 - Retrospective Case series	Proximal femoral resection with interposition arthroplasty	13 patients (18 hips)	62%	26.6	7.4	V (13)
Ackerly (2003)	Developmental Medicine & Child Neurology (3.8)	Level 4 - Retrospective Case series	Castle	7 patients (12 hips)	71%	14.7	7.7	V (7)
De Moraes Barros Fucs (2003)	Journal of Pediatric Orthopedics (1.4)	Level 4 - Retrospective Case series	Arthrodesis	14 patients (14 hips)	36%	15.4	5.3	III (4), V (10)
Leet (2005)	Journal of Pediatric Orthopedics (1.4)	Level 3 - Retrospective Cohort study	Castle; McHale	16 patients (23 hips); 11 patients (13 hips)	N/A	19.9; 14.5	3.4; 3.4	III (1), V (26) - TOTAL
Hogan (2006)	The Bone & Joint Journal (4.9)	Level 4 - Retrospective Case series	Subtrochanteric valgus osteotomy	24 patients (31 hips)	N/A	14	3.71	N/A
Abu-Rajab (2007)	Journal of Pediatric Orthopedics B (0.9)	Level 4 - Retrospective Case series	Castle	15 patients (21 hips)	33%	16.2	3.4	V (15)
Lampropulos (2008)	Journal of Pediatric Orthopedics B (0.9)	Level 4 - Retrospective Case series	Castle	3 patients (4 hips)	33%	15	1.5	V (3)
Muthusamy (2008)	Journal of Pediatric Orthopedics (1.4)	Level 4 - Retrospective Case series	Castle	27 patients (36 hips)	48%	15.5	7.8	V (27)
Egermann (2009)	The Journal of Bone & Joint Surgery (4.7)	Level 4 - Retrospective Case series	Proximal femoral resection arthroplasty with and without autologous capping	31 patients (43 hips)	N/A	21.8	2.7	V (31)
Knaus (2009)	Acta Orthopedia (2.5)	Level 4 - Retrospective Case series	Castle	20 patients (27 hips)	60%	15	3.92	IV-V (20)
Schejbalova (2009)	International Orthopedics - SICOT (2.8)	Level 4 - Retrospective Case series	Schanz	35 patients (55 hips)	N/A	13.3	8.2	V (35)
Van Riet (2009)	Acta Orthopedia Belgica (0.6)	Level 4 - Retrospective Case series	McHale	13 patients (17 hips)	69%	21	5.75	N/A
De Moraes Barros Fucs (2011)	Journal of Pediatric Rehabilitation Medicine (0.8)	Level 4 - Retrospective Case series	Arthrodesis	19 patients (19 hips)	42%	17.4	11.1	III (6), V (13)
Agashe (2013)	Orthopedics (1.0)	Level 4 - Retrospective Case series	Schanz	11 patients (17 hips)	64%	17.8	3.1	IV (3), V (8)
Wright (2013)	Journal of Pediatric Orthopedics (1.4)	Level 3 - Retrospective Cohort study	Castle; McHale; Proximal Femoral Arthroplasty with shoulder prosthesis	12 hips; 21 hips; 11 hips; 33 patients (44 hips) total	64% overall	14.3 overall	4.1 overall	N/A
Dartnell (2014)	The Bone & Joint Journal (4.9)	Level 4 - Retrospective Case series	Castle	63 patients (79 hips)	56%	14.7	5.2	IV (1), V (62)

De Moraes Barros Fucs (2014)	Journal of Pediatric Orthopedics (1.4)	Level 4 - Retrospective Case series	Arthrodesis	21 patients (21 hips)	38%	18.4	14.5	III (7), 14 (V)
Patel (2015)	Journal of Children's Orthopedics (1.1)	Level 4 - Retrospective Case series	Proximal femoral excision with interposition myoplasty	20 patients (25 hips)	35%	22	4.5	IV (9), V (9)
Godfrey (2016)	Journal of Pediatric Orthopedics (1.4)	Level 3- Retrospective Case series	Castle; McHale	6 patients (6 hips); 20 patients (24 hips)	N/A	13; 13	5.3; 1.8	V (6); IV (1), V (11)
Harmsen (2016)	Journal of Pediatric Orthopedics B (0.9)	Level 4 - Retrospective Case series	Schanz	24 patients (27 hips)	54%	13.8	8.8	IV (11), V (13)
Hwang (2016)	The Bone & Joint Journal (4.9)	Level 3 - Retrospective Cohort study	Castle; Subtrochanteric valgus osteotomy; McHale	14 patients; 10 patients; 13 patients Total 37 patients (47 hips)	38% (TOTAL)	12.3; 13.7; 11.3	4.34; 4.5; 5.1	V (14); V (10); V (13)
Morin (2016)	Orthopedics & Traumatology: Surgery & Research (2.3)	Level 4 - Retrospective Case series	Total Hip Arthroplasty	33 patients (40 hips)	52%	19.2	5	V (33)
Silverio (2016)	Journal of Childrens Orthopedics (1.1)	Level 4 - Retrospective Case series	Proximal Femoral Arthroplasty with shoulder prosthesis	12 patients (16 hips)	75%	12.2	3.4	V (12)
Martinez (2017)	Journal of Pediatric Orthopedics (1.4)	Level 4 - Retrospective Case series	Percutaneous subtrochanteric valgus osteotomy (SVO) with external fixation	15 patients (19 hips)	53%	14.3	4.2	V (15)
Chan (2019)	Journal of Pediatric Orthopedics B (0.9)	Level 3 - Retrospective Cohort study	McHale; Castle; Girdlestone; Schanz	34 patients (36 hips); 20 patients (24 hips); 3 patients (4 hips); 5 patients (5 hips)	65%; 70%; 67%; 40%	13.4; 14.8; 13.3; 14.2	2.8; 2.8; 1.0; 1.2	IV (1), V (61) (TOTAL)
Davis (2019)	Journal of Pediatric Orthopedics (1.4)	Level 3 - Retrospective Cohort study	Castle; McHale	23 patients (35 hips); 23 patients (35 hips)	26% overall	15.5; 15.5	4.5 overall	IV (2), V (21) overall
Hess (2019)	Journal of Pediatric Orthopedics (1.4)	Level 4 - Retrospective Case series	Proximal Femoral Resection	20 patients (25 hips)	N/A	13.8	2.9	N/A
Bauer (2020)	Techniques in Orthopedics (0.2)	Level 4 - Retrospective Case series	Trochanteric Sparing PFR	13 patients (17 hips)	38%	12.9	3.3	IV (2), V (11)
Horsch (2021)	Children (2.0)	Level 3 - Retrospective Cohort study	Castle with no cap; Castle with a cap	15 patients (25 hips); 23 patients (30 hips)	60%; 61%	25.93; 26.74	2.7; 2.05	IV(2), V(13); IV (5), V (18)
Koch (2021)	Frontiers in Neurology (2.7)	Level 3 - Retrospective Cohort study	Proximal Femoral Arthroplasty with shoulder prosthesis; Valgus Subtrochanteric osteotomy; Castle	20 patients (24 hips); 22 patients (24 hips); 10 patients (15 hips)	60%; 64%; 50%	14.2; 13.5; 12.9	Min 2 years	IV (2), V (18), IV (8), V (14), V (10)
Duporté (2022)	Orthopedics & Traumatology: Surgery & Research (2.3)	Level 4 - Retrospective Case series	Trochanteric Sparing PFR	3 patients (3 hips)	67%	17	1	V (3)
Doyle (2023)	The Surgeon (1.9)	Level 4 - Retrospective Case series	Proximal Femoral Arthroplasty with shoulder prosthesis	11 patients (11 hips)	36%	17.5	3.8	IV (4), V (7)
Marowsky (2024)	Journal of Pediatric Orthopedics (1.4)	Level 4 - Retrospective Case series	McHale	52 patients (65 hips)	73%	13.5	1.5	N/A
Yamada (2024)	International Orthopedics (2.0)	Level 4 - Retrospective Case series	Castle	41 patients (70 hips)	44%	13.2	6.4	V (41)

Appendix 1 Table 1B. Perioperative Results

Detailed perioperative outcomes for each study, including intraoperative complications, transfusion requirements, infections, respiratory issues, and other immediate postoperative events.

First Author (Year)	Surgery Type	Blood loss	Patients (hips)	Length of surgery	Length of Stay	Other/complications
De Moraes Barros Fucs (2003)	Arthrodesis	N/A	14 patients (14 hips)	N/A	N/A	One accidental femoral vein laceration during muscle release, 1 femur fracture, 1 sacral skin breakdown
De Moraes Barros Fucs (2011)	Arthrodesis	N/A	19 patients (19 hips)	N/A	N/A	1 skin breakdown, 1 femoral vein laceration, 2 femoral fractures
De Moraes Barros Fucs (2014)	Arthrodesis	N/A	21 patients (21 hips)	N/A	N/A	1 skin breakdown, 1 femoral vein laceration, 2 femoral fractures
Root (1986)	Arthrodesis Arthroplasty	Arthrodesis: Average 1450 mL; Arthroplasty: N/A	8 patients (8 hips); 10 patients (10 hips)	Arthrodesis: Average 220 minutes; Arthroplasty: N/A	Arthrodesis: Average 4 weeks; Arthroplasty: N/A	Arthrodesis: transfusion syndrome (1); Arthroplasty: UTI (1)
Doyle (2023)	Arthroplasty	N/A	11 patients (11 hips)	Mean 52 minutes	4.5 days mean (excluded pt who went to ICU for respiratory infection)	1 femoral fracture, which did not require surgical intervention and placement of a PEG tube for nutritional support
Gabos (1999)	Arthroplasty	Mean 964mL (range 600-5100mL)	11 patients (14 hips)	Mean 191 min (range 135 315 min)	N/A	1 broken reamer tip in canal. Two patients sustained femoral fractures postoperatively.
Morin (2016)	Arthroplasty	Mean 463 ml (range, 45-1520 ml)	33 patients (40 hips)	Average 200 min (range, 110-300 min).	N/A	Blood transfusion (18), respiratory distress due to aspiration requiring ICU admission (1), requiring intensive care complicated by respiratory infection, mesenteric artery syndrome (1), sepsis (2)
Silverio (2016)	Arthroplasty	155 ml (range 10-300 ml)	12 patients (16 hips)	N/A	N/A	Pneumonia (1), prolonged intubation (1), and postoperative anemia (3).

Abu-Rajab (2007)	Excision	N/A	15 patients (21 hips)	N/A	N/A	Two patients required transfusion, four had difficulties with reflux/vomiting during traction
Ackerly (2003)	Excision	N/A	7 patients (12 hips)	N/A	N/A	Cellulitis around IV site in one patient, pin tract infection in one patient, loosening of traction pin in four patients, small superficial area of skin necrosis due to cast pressure, non-displaced supracondylar femur fracture 3 months postop
Agashe (2013)	Excision	Mean 63 mL (range 50-100 mL)	11 patients (17 hips)	Mean 94.5 minutes (range 60-120 minutes)	Mean 10.09 days (range 4-14 days)	N/A
Barrie (1996)	Excision	N/A	6 patients (7 hips)	N/A	N/A	1 increased pain, 2 temporary increase in spasm
Bauer (2020)	Excision	Median 100 mL (95% CI, 56-275 mL)	13 patients (17 hips)	N/A	Median 3.0 days (95% CI, 2.4-5.1 days)	2 patients required blood transfusion, 1 patient cellulitis
Baxter (1989)	Excision	Mean 450 mL	4 patients (5 hips)	Mean 90 min	N/A	1 patient with L obturator neuroma and 1 patient with R heel pressure sore
Castle (1978)	Excision	N/A	12 patients (14 hips)	N/A	N/A	N/A
Chan (2019)	Excision	Mean 174 ml (range 25-700 ml)	62 patients (69 hips)	Mean 147 min (range 65-422 min)	Mean 3.8 days (range 1-18)	McHale: 5/34 (15%) required transfusion Castle: 1/20 (5%) Girdlestone: 0/3 (0%) Schanz: 0/5 (0%)
Dartnell (2014)	Excision	N/A	63 patients (79 hips)	N/A	Mean 4.1 days (range 2-7)	4 wound infections (1 requiring debridement)
Davis (2019)	Excision	N/A	46 patients (70 hips)	N/A	N/A	No radiation-associated complications reported
Duport (2022)	Excision	N/A	3 patients (3 hips)	N/A	N/A	One patient developed sub-trochanteric calcification

Egermann (2009)	Excision	N/A	31 patients (43 hips)	N/A	N/A	1 superficial wound infection
Godfrey (2016)	Excision	161.7 mL (50-700 mL)	26 patients (30 hips)	148.1 min (75-233 min)	3.0 days (1-7 days)	N/A
Harmsen (2016)	Excision	N/A	24 patients (27 hips)	127.6 min (± 48.1)	5.9 days (± 2.3)	One case of superficial infection, one of infection of the osteosynthesis material (OSM) and in two cases the OSM was dysfunctional
Hess (2019)	Excision	N/A	20 patients (25 hips)	N/A	N/A	N/A
Hoffer (1972)	Excision	N/A	2 patients (4 hips)	N/A	N/A	N/A
Hogan	Excision	N/A	24 patients (31 hips)	N/A	3.5 days average	UTI (1), pneumonia (2), wound infection (2), seroma needing drainage (2), intubation due to pneumonia (1)
Horsch (2021)	Excision	N/A	38 patients (55 hips)	N/A	N/A	Clavien-Dindo Classification: In the FHR group, 28% had no complications (grade 0), 28% had grade II, 4% had grade II, 20% had grade III, and none had grade IV complications. FCP group had more complications: Grade 0: 13.3%, Grade 1: 33.3%, Grade 2: 10%, Grade 3: 16.7%, and Grade: 43.3%
Hwang (2016)	Excision	N/A	37 patients (47 hips)	N/A	N/A	N/A
Knaus (2009)	Excision	N/A	20 patients (27 hips)	N/A	N/A	2 patients; gastric ulcer/loss of appetite/need of gastrostomy (1), DVT (1)
Lampropulos (2008)	Excision	N/A	3 patients (4 hips)	N/A	N/A	N/A

Leet (2005)	Excision	484 mL (range 100-2,000 mL)	27 patients (36 hips)	3.55 hours (1.1–5.3 hours)	5.5 days (2–27 days)	Wound dehiscence (4), skin breakdown in an area other than the incision site (3), pneumonia (1). Overall complication rate 56%; FHRT had 14 of the 16 complications (88%) compared with only 3 of the 11 (27%) complications for McHale.
Marowsky (2024)	Excision	N/A	52 patients (65 hips)	Mean operative time 178.4 minutes (range: 45 to 380 min)	11.2 days average (range: 5 to 30 d).	22 total complications in 65 McHale procedures (33.9%).UTI(2), pneumonia (1), ICU transfer (2), wound infections (8)
Martinez (2017)	Excision	Average 49mL (range 10 to 250 mL)	15 patients (19 hips)	N/A	2.77 days average (range, 1 to 8d)	15 total complications; Pin site infection (4), Skin breakdown (1), Ulcers (5), Pneumonia (2), **refracture (1),** persistent pain (1),
McHale (1990)	Excision	N/A	5 patients (6 hips)	N/A	N/A	N/A
Muthusamy (2008)	Excision	N/A	27 patients (36 hips)	N/A	N/A	N/A
Patel (2015)	Excision	N/A	20 patients (25 hips)	N/A	13 days mean (range: 3 to 35d)	Chest infection (2), superficial wound infection (1), constipation (1)
Schejbalova (2009)	Excision	N/A	35 patients (55 hips)	N/A	N/A	N/A
Van Riet (2009)	Excision	N/A	13 patients (17 hips)	N/A	N/A	Severe pulmonary infection with admission to ICU for 2 months with further development of fibrous ankylosis of the hip (1)
Widmann (1999)	Excision	N/A	13 patients (18 hips)	N/A	N/A	pneumonia (2), decubitus ulceration (4)
Yamada (2024)	Excision	N/A	41 patients (70 hips)	N/A	N/A	N/A

Koch (2021)	Excision Arthroplasty	N/A	32 patients (39 hips); 20 patients (24 hips)	N/A	N/A	N/A	
Koffman (1981)	Excision Arthroplasty	N/A	6 patients (10 hips); 2 patients (3 hips)	N/A	N/A	N/A	
Wright (2013)	Excision Arthroplasty	N/A	26 patients (33 hips); 11 patients (11 hips)	N/A	N/A	N/A	

Appendix 1 Table 1C. Postoperative Functional Outcomes

Reported mean follow-up time and post-surgical outcomes. Outcomes include pain relief, sitting tolerance, ambulation potential, and range of motion, stratified by surgical technique.

First Author (Year)	Surgery Type	Mean Time to Follow up (y)	Pain	Sitting time	Ambulation	Range of motion
De Moraes Barros Fucs (2003)	Arthrodesis	5.3	All patients had pain relief	5 of 7 bed-ridden patients improved to sitters 2 of 3 sitters became household ambulators	1 household ambulator improved to community ambulator 3 community ambulators maintained status	N/A
De Moraes Barros Fucs (2011)	Arthrodesis	11.1	All patients had pain relief	Improved in some patients	2 improved from sitters to household walkers, 1 household walker improved to community walker, 1 household walker became sitter	N/A
De Moraes Barros Fucs (2014)	Arthrodesis	14.5	All patients had pain relief	6 bedridden patients improved to sitters	1 sitter returned to community ambulation, 4 community ambulators maintained status	N/A
Root (1986)	Arthrodesis Arthroplasty	6.75	Arthrodesis: 6/8 hips had pain relief immediately without revisions; THA: 14/15 patients had pain relief	Improved in both groups	Arthrodesis: 3 patients able to walk again, 4 maintained walking ability; THA: 8 able to ambulate	Arthrodesis: improved, not quantified; THA: abduction and rotation improved in all patients, not quantified
Doyle (2023)	Arthroplasty	3.8	5 excellent, 4 good, 2 fair results	Not specifically measured, but improvements in seating reported by caregivers	All non-ambulatory	Reported as 5 excellent or 6 good
Gabos (1999)	Arthroplasty	5	10 of 11 patients had complete relief of hip pain	Improved in all patients, 5 achieved unlimited sitting tolerance	All non-ambulatory	N/A
Morin (2016)	Arthroplasty	5	Permanent pain (0), sitting (1), transfer (0), perineal care (2); all statistically significant (p<0.05)	N/A	No significant improvement (P>0.05)	Flexion >80 degrees showed significant improvement (P<0.05)
Silverio (2016)	Arthroplasty	3.4	Excellent (9), good (0), fair (4), poor (3).	Average change in sitting tolerance was 6.6/10	All remained non-ambulatory	Excellent (6), good (3), fair (6), poor (1)

			Average change in pain was 8.2/10			
Abu-Rajab (2007)	Excision	3.4	18/20 hips with pre-op pain had significant improvement or complete resolution	All 12 hips with seating difficulties improved post-op	All non-ambulatory	N/A
Ackerly (2003)	Excision	7.7	5/7 pts (9 hips) had no pain, 2 pts (3 hips) had slight pain but no medication required	All patients able to sit for at least 4-5 hours	All non-ambulatory	6/7 patients maintained functional ROM with near full extension to 90 of<br>flexion and at least 30 of abduction
Agashe (2013)	Excision	3.1	Visual analog pain score improved from mean 8.36 pre-op to 3.27 post-op ($p<0.0001$)	Improved from 30 minutes pre-op to 120 minutes post-op	All non-ambulatory	Adduction deformity converted to abduction deformity (mean 16Â°), flexion deformity decreased to mean 20Â°
Barrie (1996)	Excision	1.4	2 - none, 1 -occasional, 2 - frequent, 1 -constant	N/A	All non-ambulatory	Mean total range of movement (deg) 118.3 +30.5
Bauer (2020)	Excision	3.3	12/13 patients had complete pain relief	All patients had improved sitting tolerance	One patient regained limited household ambulation ability	All patients regained functional hip range of motion
Baxter (1989)	Excision	N/A	All pain-free	All comfortable sitting pain free	All non-ambulatory	Marked improvement in range of motion postoperatively
Castle (1978)	Excision	2	All hips pain-free post-op	All patients able to sit with ease post-op, some loss of motion in most patients after 6 months	All non-ambulatory	Some loss of motion in most patients after 6 months - 25% loss in 2 cases, 10-20% loss in 7 cases, no loss in 3 cases
Chan (2019)	Excision	2.59 (0.5 - 10.42 years)	Pain improved in: McHale: 25/34 (74%) Castle: 14/20 (70%) Girdlestone: 3/3 (100%) Schanz: 3/5 (60%)	N/A	All non-ambulatory	N/A
Dartnell (2014)	Excision	5.2	71 hips (89.6%) reported to be pain free or have mild pain post-	One child with pre-operative standing lost this ability post-surgery	All non-ambulatory	N/A

			operatively			
Davis (2019)	Excision	4.5 overall	Duration of postoperative pain lower in patients without HO (1.7 vs 14.3 months p=0.017)	N/A	All non-ambulatory	N/A
Duport (2022)	Excision	1	All patients had absence of pain post-operatively	Improved sitting and perineal care reported	All non-ambulatory	N/A
Egermann (2009)	Excision	2.7	Improved in all patients	Improved in all patients	All non-ambulatory	N/A
Godfrey (2016)	Excision	2.44 (0.08 - 7.5)	66% (50-85%) reported improved	69% reported improved	N/A	N/A
Harmsen (2016)	Excision	8.8	Mean NCCPC-PV score of 14.8 (similar to CP patients without surgical intervention)	N/A	All non-ambulatory	N/A
Hess (2019)	Excision	2.9	Symptomatic HO (defined as new pain, progressive pain, or increased limited range of motion) occurred in 45.5% of pre-op and 35.7% of post-op patients	N/A	N/A	N/A
Hoffer (1972)	Excision	N/A	N/A	Patient 1: 6 hours postoperatively with- out pain. Patient 2: 8 hours propped in trunk-brace and thigh-cuffs in wheelchair.	All non-ambulatory	pt 1. 30" flexion contracture with further flexion to 120': 25" abduction range. pt2. Painless (L) hip, 20" flexion contracture, 100" of hip flexion
Hogan	Excision	3.71	Resolution of pain in all but 1	2 hours (11), others >8 hours,	N/A	N/A
Horsch (2021)	Excision	2.3	11 patients	N/A	no change from baseline specified for non-ambulatory and minimal ambulatory patients	N/A
Hwang (2016)	Excision	4.67 (2 - 9.5)	Significant improvements in pain and hurt ($p < 0.001$) according	N/A	All non-ambulatory	Significant improvements in PedsQL scores occurred in movement

			to PedsQL and CPPCHILD			and balance (p < 0.001)
Knaus (2009)	Excision	3.92	Complete relief (8), improvement (7), no change (1), worse (1), no pain before or after (1)	N/A	N/A	Mean pelvic obliquity was 11 (0–33)° before surgery and 6.4 (0–15)° at follow-up (p = 0.1)
Lampropulos (2008)	Excision	1.5	All 3 patients had improvement in pain relief.		All non-ambulatory	Physical therapists reported an improvement in the range of motion of all four hips.
Leet (2005)	Excision	3.4 years (5 weeks to 22 years)	FHRT postoperative pain averaged 2.9; McHale group postoperative pain averaged 4.8 after surgery. Not statistically significant. Pain free status with sitting tolerance achieved at and average of 2.5 months	Increased by 3 hours on average	Overall functional gains after surgical intervention (5), including performing standing transfers (2) and using a standing frame (2)	N/A
Marowsky (2024)	Excision	1.5	3.31/10 mean, statistically significant (p<0.001)	199 minutes, not statistically significant	No statistically significant improvement in mobility	No statistically significant improvement to range of motion
Martinez (2017)	Excision	4.2	CPCHILD questionnaire questions assessing pain showed improvement (P = 0.004).	Improved	N/A	Average 24 degrees (P < 0.0001)
McHale (1990)	Excision	3.1	Pain free ROM and sitting	All patients able to sit 3-4 hour intervals	N/A	90 degree flexion, 30 degree abduction, 5 degree extension
Muthusamy (2008)	Excision	7.8	Grade 1 (26), Grade 2 (2), Grade 3 (2); There was a significant (P G 0.05) decrease of 2 grades in the overall pain score.	Improved by 4.4 hours	N/A	Significant improvement (P<0.05) in adduction, abduction, flexion and extension. No significant improvement (P>0.05) in internal and external rotation

Patel (2015)	Excision	4.5	Visual analog pain score mean 2.8/10 (p<0.001)	65% could sit >3 hours, all patients could sit >1 hour	All remained non-ambulatory	Hip flexion 0-90°, abduction to at least 20°, free rotation
Schejbalova (2009)	Excision	8.2	Pain relief (51 patients), persistent transient hip pain (4)	Improved in all patients	All remained non-ambulatory	Abduction of 30–35° (49 hips), improved range of movement with final abduction >30° (6)
Van Riet (2009)	Excision	5.75	Pain free (9), periodically (2), painful left hip (1), improvement in pain (2)	Improvement (7), no change (10)	N/A	Hip flexion of at least 90° (14), flexion 70° (3); full extension (11) extension deficit of 10° to 30° (4), fixed in 90° of flexion (2); average abduction of 35° (16), limited abduction (1)
Widmann (1999)	Excision	7.4	Improved in pain (11), incomplete pain relief (2); non-narcotic pain med use average 0.6 pills, narcotic pain med use average 0.0 pills	Average 534 minutes, sitting upright with maximal recline <15 degrees (12), need 30 degree angle (1)	All remained non-ambulatory	Hip flexion improved to mean 107 degrees, hip extension improved to mean 12 degrees, hip abduction improved to mean 44 degrees, hip adduction improved to mean 52 degrees, internal rotation improved to mean 29 degrees, no significant improvement in external rotation
Yamada (2024)	Excision	6.4	Pain free (37)	Sitting in a wheelchair: very easy (37), a little difficult (1), difficult (2), impossible (1)	All remained non-ambulatory	N/A

Koch (2021)	Excision Arthroplasty	Min 2 years	A decline in pain complaints in all four therapeutic groups was observed. The PFIA, SO, and HR groups recorded a pain relief from 4.93 (1–10) to 0.93 (0–5), 6.22 (3–10) to 0.59 (0–6), and 5.42 (2–10) to 2.13 (0–5) in the NRS score, respectively (p < 0.001). The pain score was reduced comparably lying position, perineal care, and sitting position. The reduction difference was statistically significant in all cases except patients after proximal femoral resection, where the change in pain at the lying position was not statistically significant (p = 0.15). No statistical difference between GMFCS groups. Correlated strongly with age, older patients had less pain relief.	Sitting improved in sitting position, p <0.001 (all patients)	All non-ambulatory	N/A
Koffman (1981)	Excision Arthroplasty	8; N/A	PFHR: improvement of pain in 5/6 patients ; THA: resolved in female, resolved then deteriorated in male	PFHR: 5/6 patients improved to 7.5 hours, 6th patient improved to this result post re-resection 4.5 years later ; THA: Female (10 hours), male (5 hours)	All non-ambulatory	PFHR: hip flexion range and total flexion-extension range of the hip motion improved in 6 hips, unchanged in 2 hips, decreased in 2 hips; THA: Female: N/A, male: 50 degree flexion-extension in the early months post-op but 9 months post of his ROM decreased significantly due to excessive HO.

Wright (2013)	Excision Arthroplasty	4.1	SVO: excellent (52.4%), good (14.3%), fair (9.5%), poor (23.8%); PFRA: excellent (31.6%), good (36.8%), fair (5.3%), poor (26.3%); PFIA: excellent (44.4%), good (33.3%), fair (0.0%), poor (22.2%); no statistical significance between groups	N/A	N/A	SVO: excellent (57.1%), good (23.8%), fair (14.3%), poor (4.8%); PFRA: excellent (42.1%), good (47.4%), fair (10.5%), poor (0.0%); PFIA: excellent (61.1%), good (27.8%), fair (11.1%), poor (0.0%); no statistical significance between groups

Appendix 1 Table 1D. Postoperative Clinical and Radiographic Outcomes - Late postoperative outcomes, including heterotopic ossification, scoliosis progression, femoral migration, mechanical failures or need for revision surgery, caregiver-reported satisfaction, and patient mortality where noted.

First Author (Year)	Surgery Type	Heterotopic Ossification	Scoliosis	Leg length discrepancy or femoral migration	Surgical failure/Revisions/mechanical complications	Caretaker reported outcomes	Deaths
De Moraes Barros Fucs (2003)	Arthrodesis	N/A	2 patients remained bed-ridden due to severe scoliosis	N/A	3 patients (21.4%) developed pseudarthrosis requiring reoperation	N/A	N/A
De Moraes Barros Fucs (2011)	Arthrodesis	N/A	3 patients remained bedridden due to severe scoliosis	N/A	4 patients (21%) developed pseudarthrosis requiring reoperation	Patient satisfaction reported for all cases	N/A
De Moraes Barros Fucs (2014)	Arthrodesis	N/A	3 patients remained bedridden due to severe scoliosis	N/A	4 patients (19%) developed pseudarthrosis requiring reoperation	Patient/caregiver satisfaction reported for all cases	N/A
Root (1986)	Arthrodesis Arthroplasty	Arthrodesis: N/A; THA: Grade 2 (4), Grade 3 (4), total 53% incidence	N/A	Arthrodesis: N/A, THA: migration of greater trochanter (1)	arthrodesis: pseudoarthrosis fixed with iliac bone graft (1), pseudoarthrosis revised with THA (1), supracondylar fracture (1), painful bursa fixed with screw removal (1); THA: recurrent dislocation managed with revision (1), trochanteric bursitis (3), revision with replacement of femoral component (1)	N/A	N/A
Doyle (2023)	Arthroplasty	None observed in follow-up radiographs	N/A	2 patients showed proximal femoral migration	2 patients required implant removal due to ongoing pain	The majority of patients reported being satisfied or very satisfied with outcomes across all categories, with particularly high satisfaction in seating and perineal care. Pain management and transfers showed mixed results, while overall satisfaction was predominantly positive.	N/A
Gabos (1999)	Arthroplasty	5 patients developed heterotopic ossification	N/A	N/A	No patients required additional hip surgery, except one who needed bilateral abductor and hamstring releases	10 of 11 caretakers would recommend the procedure	N/A
Morin (2016)	Arthroplasty	N/A	23 patients; 16 cases of spinal fusion prior to THA	N/A	Six mechanical complications required revision surgery: femoral loosening (1), delayed femoral osteotomy consolidation managed by decortication, graft and internal fixation (2), greater trochanter detachment (1), osteoma managed by resection (1), disassembly between the prosthetic femoral head and the Morse cone (1); 5 mechanism complications did not require revision: acetabular loosening (1), osteoma (1), proximal metaphyseal fracture (2), lateral cortex resorption (1)	100% reported alleviation of pain, 14% reported no improvement in sitting comfort, 34% reported perineal care was not made easier	3 deaths
Silverio (2016)	Arthroplasty	Average Brooker Grading of 1.4 (6 patients)	N/A	38% average prosthesis migration	Revision secondary to prominent prostheses (1), revision after developing severe pain and osteolysis (1), periprosthetic fracture (1 patient),	Average change in perineal hygiene was 6/10, average satisfaction was 8.6/10, average recommendation was 8.5/10	N/A
Abu-Rajab (2007)	Excision	12/21 hips (57%) developed heterotopic ossification (11 grade 1 1 grade 2)	N/A	19/21 hips had proximal femur at level of acetabulum post-op, 2/21 below acetabulum	1 patient required stump revision at 9 months for proximal migration	All 8 hips with hygiene difficulties improved post-op	N/A
Ackerly (2003)	Excision	All developed some HO radiographically- 7 hips type I, 1 hip type II changes, 4 hips diffused type III - none interfered with clinical outcome	2 required surgery for scoliosis post hip surgery	All hips demonstrated some degree of proximal femoral migration- 4 hips grade I, 3 hips grade II, 5 hips grade III only 1 pt grade III slightly painful	0 surgical revisions required	Improved comfort, sitting, hygiene and perineal care, improved function, time to achieve max benefit was 6-12 month (range 3 to 18 months)	One died 4.5 years after procedure
Agashe (2013)	Excision	N/A	N/A	N/A	11 complications in 7 patients - 6 minor (pin tract infections), 5 major (delayed consolidation, skin ulceration, pneumonia, abduction contracture)	All caregivers satisfied, reported improved transfers and perineal care	N/A
Barrie (1996)	Excision	N/A	N/A	N/A	1 failed excision arthroplasty on child with good result on other hip, continues to have disabling pain	Overall opinion: 2- much better, 2- a bit better, 1- no different, 1- much worse	N/A
Bauer (2020)	Excision	One patient developed clinically insignificant Brooker stage 3 heterotopic ossification, no other significant HO	N/A	Median migration 12.4 mm (95% CI, 7-19 mm)	1 patient required revision for failed soft tissue envelope	10/11 caregivers would recommend the procedure at final follow-up	N/A
Baxter (1989)	Excision	No myositis ossificans or periarticular calcification observed	N/A	Minimal proximal femoral migration	No further surgery on any of 5 hips	Dramatic improvement in patients' general well-being, ease of handling/mobilizing, and perineal care	N/A
Castle (1978)	Excision	N/A	N/A	N/A	N/A	All patients easily cared for in bed post-op, no skin maceration/ulceration, all able to sit with ease	N/A
Chan (2019)	Excision	Heterotopic ossification: McHale: 7/34 (21%) Castle: 11/20 (55%) Girdlestone: 3/3 (100%) Schanz: 0/5 (0%)	N/A	Proximal femoral migration: McHale: 13/34 (38%) Castle: 6/20 (30%) Girdlestone: 2/3 (67%) Schanz: 0/5 (0%)	Revision surgery required: McHale: 14/34 (41%) Castle: 3/20 (15%) Girdlestone: 1/3 (33%) Schanz: 0/5 (0%)	N/A	N/A
Dartnell (2014)	Excision	16 hips (20.2%) showed radiographic evidence of heterotopic ossification	N/A	N/A	4 patients (6.5%) required further resection for persistent pain	59 patients (94%) reported improvements in perineal hygiene and transfers	N/A
Davis (2019)	Excision	HO developed in 35% of radiated hips vs 83% of non-radiated hips (p=0.015)	N/A	N/A	Reoperation for HO required in 11% of non-radiated hips vs 0% of radiated hips	N/A	N/A
Duport (2022)	Excision	One patient had asymptomatic heterotopic (McCarthy type 3) ossification.	None reported	N/A	N/A	All caregivers reported being very satisfied	N/A
Egermann (2009)	Excision	2/18 hips	N/A	Proximal migration in 6/18 hips	0	Improved nursing care in all patients	0
Godfrey (2016)	Excision	6.25% in modified McHale group vs 50% in standard McHale	N/A	17.2 mm (0-46 mm)	3/16 modified McHale patients required hardware removal, 1 required revision for insufficient resection/valgus	Pain improved? 85% Perineal care improved?85% Sitting tolerance improved? 69% Recommend this procedure? 69%	N/A
Harmsen (2016)	Excision	N/A	N/A	N/A	4 reoperations (1 proximal femoral head resection, 3 adjustments of osteosynthesis material)	12 caregivers expressed that their expectations were met, and 11 expressed that their expectations were not met	2 patients deceased (unrelated to surgery)
Hess (2019)	Excision	Radiographic evidence of HO was found in all 25 hips	N/A	N/A	N/A	N/A	N/A
Hoffer (1972)	Excision	N/A	None reported	N/A	N/A	Perineal care improved, pain improved	N/A

Hogan	Excision		2 patients	2 patients – no surgical repair	N/A	3 patients required revision because of hardware failure. There was one intraoperative femoral fracture. 3 patients had screw removals due to persistent pain/development of pain	14 caregivers stated that hip motion had been improved, and pain had been decreased after the surgery. 14 stated that they would have the surgery done again if they had the decision to make.	N/A
Horsch (2021)	Excision		Grade II (FHR 36% vs. FCP 53.3%), Grade III (FHR vs FCP at 8% and 10%)	N/A	Telescoping mentioned here: FHR - 18.86% (11), FCP 31.99, no significant difference between groups, this is proximal femoral migration.	FCP group - 4 needed surgery again (2 due to pain, 1 due to telescoping, 1 due to cerclage-insufficiency), FHR group - 1 needed surgery again due to pain. 1 patient who underwent proximal FHR had a re-resection surgery.	N/A	N/A
Hwang (2016)	Excision		6 patients (43%) and consisted of type I heterotopic ossification	N/A	3 patients had asymptomatic migration of the proximal femur.	2 patients (20%) developed bursitis over the femoral head in the gluteal region treated by excision of the femoral head. 4 patients (31%) needed surgical revisions with hardware. 3 patients had pain over the plate, which resolved when the plate was removed. A plate broke in 1 patient, which was treated by revision using two plates.	According to both the PedsQL parent proxy and CPCHILD surgery which is also done by caretakers these were the ones that were statistically significant: 1. personal care/activities of daily living 2. positioning, transferring and mobility 3. comfort and emotions 4. Health 5. total score for overall CPCHILD survey<br><br>PedsQL:<br>1. movement and balance<br>2. pain and hurt	N/A
Knaus (2009)	Excision		Type 1 (3), type 2 (6), and type 3 (12)	N/A	Proximal femoral migration mean 2.1cm. Mean migration from the first postoperative day to the last follow-up was mean 2.9 cm. Total migration on the dislocated side from preoperatively to the last follow-up was mean 4.8 cm. Total migration was mean 8.1cm in the located hips that had undergone femoral resection.	Prolonged pain lasting more than 3 months and up to 6 months postoperatively in 7 patients. 1 patient was reoperated with resection of the bone spike causing complaints, but she died 11 days after surgery.	Satisfied (14), dissatisfied (3), uncertain (2); dissatisfied caretakers were all of those where the patient died. Perineal care improved in all but 1 patient	During the follow-up period 3 patients died; unknown causes (1), pneumonia (1), respiratory insufficiency (1)
Lampropulos (2008)	Excision		All (3) patients developed minimal HO with no effect on clinical outcome	N/A	3 hips: proximal femur was below the level of the acetabulum; 1 hip: proximal femur migration above the level of the acetabulum	1 patient required extensive femoral resection due to continued pain and femur migration about acetabulum.	Clinical improvement in all patients with respect to perineal care	N/A
Leet (2005)	Excision		Heterotopic ossification (5 patients), only seen in patients who underwent FHRT	N/A	Mean 1.7 cm	Hardware failure (2 patients), and infected implants (1 patient).	8/15 (53%), would have the surgery again, 11/15 (73%) would recommend the surgery to someone else. 2 caretakers reported worse outcomes, 2 caretakers stated no improvement (one child in each of the surgical groups). Average overall postoperative satisfaction score was 9/10 (FHRT) and 7.7 (McHale), not a statistically significant difference (P = 0.44).	McHale (1), perforated viscus unrelated to surgery
Marowsky (2024)	Excision		Twenty-six patients had no HTOs (59.1%), stage 1 (12), stage 2 HTO (4), stage 3 (2),	N/A	Mean migration distance was 10.7mm	Surgical revision due to wound infection (3), surgical revision post implant removal due to hematoma (1)	85% of the caregivers would have the surgery performed on their child again and 81% of the caregivers would recommend the surgery to others. Overall, 50% reported by caretakers as having good or very good overall satisfaction	N/A
Martinez (2017)	Excision		N/A	N/A	Osteotomy angle averaged 35 degrees; Pelvic femoral shaft angle averaged 5.4 degrees; Hip abduction averaged 24 degrees	Refractures following external fixator removal (1), persistent pain resolved with PFR (1)	CPCHILD score (possible score, 10 to 50) improved from 27.2 to 16.23 (P = 0.05) as answered per caretakers	N/A
McHale (1990)	Excision		Minimal amounts (1)	4 patients, 3 underwent spinal fusion	No proximal femoral migration	Plate removal due to persistent pain (1), plate removal for spinal fusion (1)	N/A	N/A
Muthusamy (2008)	Excision		Type 1 (4), Type 2 (8), Type 3 (3)	N/A	Femoral migration (7); 6 proximal and 1 distal	N/A	77% of patients and/or caregivers reported a perceived decrease in patient irritability, 83% reported improvement in overall satisfaction after surgery	N/A
Patel (2015)	Excision		3/12 hips at follow up with minor HO	N/A	No significant proximal femoral migration	Femur protrusion from the buttocks due to failed interposition myoplasty treated with surgical revision (1)	Perineal care improved in all patients, all patients scores "satisfied" or "very satisfied"	N/A
Schejbalova (2009)	Excision		N/A	fixed lumbar hyper lordosis (14 patients)	Asymmetrical hip position (windblown hip) (1 patients)	Revision due to asymmetrical hip position (1), persistent severe pain amended by Schanz procedure (1)	N/A	N/A
Van Riet (2009)	Excision		Minimal (2)	N/A	1 patient had significant proximal femoral migration, all other patients showed minimal amounts	Failure of osteosynthesis with dislocation of the lesser trochanter causing hardware removal (1), removal of screws due to protrusion into acetabulum (1), revision proximal femoral resection due to persistent irritation (1), hardware removal to improve pain (7) causing resolution of pain in (5), femur fracture at distal screw with subsequent PFR (1); Overall hardware was removed in 11 of 17 hips and a proximal femoral resection was performed in two other hips	Easier nursing and hygiene maintenance due to decreased pain and improved mobility (10), no different in nursing care (3)	N/A
Widmann (1999)	Excision		Grade 0 (2), Grade 1 (3), Grade 2 (0), Grade 3 (10), Grade 4 (3); evidence of decrease HO with post-op radiation ($P<0.005$)	N/A	Above the acetabulum (5), at the level of the acetabulum (9), below the level of the acetabulum (4)	Ulcer requiring debridement (1), revisional resection arthroplasty due to HO (2)	Improvement in daily living; Continued difficulty with feeding (1), difficulty with pulmonary toilet (1), problems with perineal hygiene (0), decubitus ulcers (0)	N/A
Yamada (2024)	Excision		Present in all patients; Type 1 (27 hips), Type 2 (41 hips), Types 3 (2 hips)	40/41 patients	0% (7 hips), 0-100% (32 hips), 100% (27 hips), >100% (4 hips)	Tibial fracture (1), diaphyseal R femur fracture (1), left distal femur fracture (1), revision due to massive HO (4)	CPCHILD: Improved quality of life mean score 21.95, level 4 (35),	N/A
Koch (2021)	Excision	Arthroplasty	PFIA (Grade nm, 6 hips); Schanz valgus osteotomy (Grade nm, 2 hips)	N/A	N/A	2 patients required revision surgery. SO (hardware failure, 1 patient), HR (femoral stump migration).	The treatment satisfaction scores reported by the parents or caregivers were 7.41 (PFIA), 5.9 (SO), and 8.3 (HR). The proportions of parents or caregivers who would not have consented to the surgery again were 5/20 (PFIA), 8/22 (SO), and 2/10 (HR).	5 patients died at follow up
Koffman (1981)	Excision	Arthroplasty	PFHR: 100% cohort had evidence of HO, 2 hips with excessive HO, 1 required re-operation; THA: male, mass HO, female nm	N/A	N/A	PFHR: 2 patients, re-resection of proximal femur (1), insertion of prothesis due to HO (1)	N/A	1 patient died due to unrelated causes

Wright (2013)	Excision	Arthroplasty	5 patients; caused failure of PFRA	N/A	N/A	Total 14 revisions (9)	All caretakers were happy with the final results and would recommend the final surgery performed to others	N/A

Appendix 1 Table 1E: Postoperative Functional Outcomes
Reported mean follow-up time and post-surgical outcomes. Outcomes include: pain relief, sitting tolerance and caretaker reported outcomes stratified by surgical technique.

First Author (Year)	Surgery Type	Mean Time to Follow up (y)	Pain	Sitting Time	Caretaker Reported Outcomes
De Moraes Barros Fucs (2003)	Arthrodesis	5.3	All patients had pain relief	5 of 7 bed-ridden patients improved to sitters 2 of 3 sitters became household ambulators	N/A
De Moraes Barros Fucs (2011)	Arthrodesis	11.1	All patients had pain relief	Improved in some patients	Patient satisfaction reported for all cases
De Moraes Barros Fucs (2014)	Arthrodesis	14.5	All patients had pain relief	6 bedridden patients improved to sitters	Patient/caregiver satisfaction reported for all cases
Root (1986)	Arthrodesis Arthroplasty	6.75	Arthrodesis: 6/8 hips had pain relief immediately without revisions; THA: 14/15 patients had pain relief	Improved in both groups	N/A
Doyle (2023)	Arthroplasty	3.8	5 excellent, 4 good, 2 fair results	Not specifically measured, but improvements in seating reported by caregivers	The majority of patients reported being satisfied or very satisfied with outcomes across all categories, with particularly high satisfaction in seating and perineal care. Pain management and transfers showed mixed results, while overall satisfaction was predominantly positive.
Gabos (1999)	Arthroplasty	5	10 of 11 patients had complete relief of hip pain	Improved in all patients, 5 achieved unlimited sitting tolerance	10 of 11 caretakers would recommend the procedure
Morin (2016)	Arthroplasty	5	Permanent pain (0), sitting (1), transfer (0), perineal care (2); all statistically significant ($p<0.05$)	N/A	100% reported alleviation of pain, 14% reported no improvement in sitting comfort, 34% reported perineal care was not made easier
Silverio (2016)	Arthroplasty	3.4	Excellent (9), good (0), fair (4), poor (3). Average change in pain was 8.2/10	Average change in sitting tolerance was 6.6/10	Average change in perineal hygiene was 6/10, average satisfaction was 8.6/10, average recommendation was 8.5/10
Abu-Rajab (2007)	Excision	3.4	18/20 hips with pre-op pain had significant improvement	All 12 hips with seating difficulties improved	All 8 hips with hygiene difficulties improved post-op

			or complete resolution	post-op	
Ackerly (2003)	Excision	7.7	5/7 pts (9 hips) had no pain, 2 pts (3 hips) had slight pain but no medication required	All patients able to sit for at least 4-5 hours	Improved comfort, sitting, hygiene and perineal care, improved function, time to achieve max benefit was 6-12 month (range 3 to 18 months)
Agashe (2013)	Excision	3.1	Visual analog pain score improved from mean 8.36 pre-op to 3.27 post-op (p<0.0001)	Improved from 30 minutes pre-op to 120 minutes post-op	All caregivers satisfied, reported improved transfers and perineal care
Barrie (1996)	Excision	1.4	2-none, 1-occasional, 2-frequent, 1-constant	N/A	Overall opinion: 2-much better, 2-a bit better, 1-no different, 1-much worse
Bauer (2020)	Excision	3.3	12/13 patients had complete pain relief	All patients had improved sitting tolerance	10/11 caregivers would recommend the procedure at final follow-up
Baxter (1989)	Excision	N/A	All pain-free	All comfortable sitting pain free	Dramatic improvement in patients' general well-being, ease of handling/mobilizing, and perineal care
Castle (1978)	Excision	2	All hips pain-free post-op	All patients able to sit with ease post-op, some loss of motion in most patients after 6 months	All patients easily cared for in bed post-op, no skin maceration/ulceration, all able to sit with ease
Chan (2019)	Excision	2.59 (0.5 - 10.42 years)	Pain improved in:<br>McHale: 25/34 (74%)<br>Castle: 14/20 (70%)<br>Girdlestone: 3/3 (100%)<br>Schanz: 3/5 (60%)	N/A	N/A
Dartnell (2014)	Excision	5.2	71 hips (89.6%) reported to be pain free or have mild pain post-operatively	One child with pre-operative standing lost this ability post-surgery	59 patients (94%) reported improvements in perineal hygiene and transfers
Davis (2019)	Excision	4.5 overall	Duration of post-operative pain lower in patients without HO (1.7 vs 14.3 months p=0.017)	N/A	N/A
Duport (2022)	Excision	1	All patients had absence of pain post-operatively	Improved sitting and perineal care reported	All caregivers reported being very satisfied
Egermann (2009)	Excision	2.7	Improved in all patients	Improved in all patients	Improved nursing care in all patients
Godfrey (2016)	Excision	2.44 (0.08 - 7.5)	66% (50-85%) reported improved	69% reported improved	Pain improved? 85%<br>Perineal care improved? 85%<br>Sitting tolerance improved? 69%<br>Recommend this procedure? 69%
Harmsen (2016)	Excision	8.8	Mean NCCPC-PV score of 14.8 (similar to CP patients without surgical intervention)	N/A	12 caregivers expressed that their expectations were met, and 11 expressed that their expectations were not met

Hess (2019)	Excision	2.9	Symptomatic HO (defined as new pain, progressive pain, or increased limited range of motion) occurred in 45.5% of pre-op and 35.7% of post-op patients	N/A	N/A
Hoffer (1972)	Excision	N/A	N/A	Patient 1: 6 hours post-operatively without pain.<br><br>Patient 2: 8 hours propped in trunk-brace and thigh-cuffs in wheelchair.	Perineal care improved, pain improved.
Hogan (2006)	Excision	3.71	Resolution of pain in all but 1	2 hours (11), others >8 hours	14 caregivers stated that hip motion had been improved, and pain had been decreased after the surgery. 14 stated that they would have the surgery done again if they had the decision to make.
Horsch (2021)	Excision	2.3	11 patients	N/A	N/A
Hwang (2016)	Excision	4.67 (2 - 9.5)	Significant improvements in pain and hurt<br>($p < 0.001$) according to PedQL and<br>CPCHILD	N/A	According to both the PedsQL parent proxy and CPCHILD surgery which is also done by caretakers these were the ones that were statistically significant:<br><br>1. Personal care/activities of daily living<br>2. Positioning, transferring and mobility<br>3. Comfort and emotions<br>4. Health 5. total score for overall CPCHILD survey<br><br>PedQL:<br>1. Movement and balance<br>2. Pain and hurt
Knaus (2009)	Excision	3.92	Complete relief (8), improvement (7), no change (1), worse (1), no pain before or after (1)	N/A	Satisfied (14), dissatisfied (3), uncertain (2); dissatisfied caretakers were all of those where the patient died. Perineal care improved in all but 1 patient
Lampropulos (2008)	Excision	1.5	All 3 patients had improvement in pain relief	N/A	Clinical improvement in all patients with respect to perineal care
Leet (2005)	Excision	3.4 years<br>(5 weeks to 22 years)	FHRT post-operative pain averaged 2.9; McHale group post-operative pain averaged 4.8 after surgery. Not	Increased by 3 hours on average	8/15 (53%), would have the surgery again, 11/15 (73%) would recommend the surgery to someone else. 2 caretakers

			statistically significant. Pain free status with sitting tolerance achieved at an average of 2.5 months		reported worse outcomes, 2 caretakers stated no improvement (one child in each of the surgical groups). Average overall post-operative satisfaction score was 9/10 (FHRT) and 7.7 (McHale), not a statistically significant difference (P = 0.44)
Marowsky (2024)	Excision	1.5	3.31/10 mean, statistically significant (p<0.001)	199 minutes, not statistically significant	85% of the caregivers would have the surgery performed on their child again and 81% of the caregivers would recommend the surgery to others. Overall, 50% reported by caretakers as having good or very good overall satisfaction
Martinez (2017)	Excision	4.2	CPCHILD questionnaire questions assessing pain showed improvement (p = 0.004).	Improved	CPCHILD score (possible score, 10 to 50) improved from 27.2 to 16.23 (P = 0.05) as answered per caretakers
McHale (1990)	Excision	3.1	Pain free ROM and sitting	All patients able to sit 3-4 hour intervals	N/A
Muthusamy (2008)	Excision	7.8	Grade 1 (26), Grade 2 (2), Grade 3 (2); There was a significant (P G 0.05) decrease of 2 grades in the overall pain score.	Improved by 4.4 hours	77% of patients and/or caregivers reported a perceived decrease in patient irritability, 83% reported improvement in overall satisfaction after surgery
Patel (2015)	Excision	4.5	Visual analog pain score mean 2.8/10 (p<0.001)	65% could sit >3 hours, all patients could sit >1 hour	Perineal care improved in all patients, all patients scores "satisfied" or "very satisfied"
Schejbalova (2009)	Excision	8.2	Pain relief (51 patients), persistent transient hip pain (4)	Improved in all patients	N/A
Van Riet (2009)	Excision	5.75	Pain free (9), periodically (2), painful left hip (1), improvement in pain (2)	Improvement (7), no change (10)	Easier nursing and hygiene maintenance due to decreased pain and improved mobility (10), no different in nursing care (3)
Widmann (1999)	Excision	7.4	Improved in pain (11), incomplete pain relief (2); non-narcotic pain med use average 0.6 pills, narcotic pain med use average 0.0 pills	Average 534 minutes, sitting upright with maximal recline <15 degrees (12), need 30 degree angle (1)	Improvement in daily living; Continued difficulty with feeding (1), difficulty with pulmonary toilet (1), problems with perineal hygiene (0), decubitus ulcers (0)
Yamada (2024)	Excision	6.4	Pain free (37)	Sitting in a wheelchair: very easy (37), a little difficult (1), difficult (2), impossible (1)	CPCHILD: Improved quality of life mean score 21.95, level 4 (35)
Koch (2021)	Excision	Min. 2 years	A decline in pain complaints	Sitting improved in sitting	The treatment satisfaction scores

	Arthroplasty		in all four therapeutic groups was observed. The PFIA, SO, and HR groups recorded a pain relief from 4.93 (1–10) to 0.93 (0–5), 6.22 (3–10) to 0.59 (0–6), and 5.42 (2–10) to 2.13 (0–5) in the NRS score, respectively ($p < 0.001$). The pain score was reduced comparably lying position, perineal care, and sitting position. The reduction difference was statistically significant in all cases except patients after proximal femoral resection, where the change in pain at the lying position was not statistically significant ($p = 0.15$). No statistical difference between GMFCS groups. Correlated strongly with age, older patients had less pain relief.	position, $p < 0.001$ (all patients)	reported by the parents or caregivers were 7.41 (PFIA), 5.9 (SO), and 8.3 (HR). The proportions of parents or caregivers who would not have consented to the surgery again were 5/20 (PFIA), 8/22 (SO), and 2/10 (HR)
Koffman (1981)	Excision Arthroplasty	8; N/A	PFHR: improvement of pain in 5/6 patients ; THA: resolved in female, resolved then deteriorated in male	PFHR: 5/6 patients improved to 7.5 hours, 6th patient improved to this result post re-resection 4.5 years later; THA: female (10 hours), male (5 hours)	N/A
Wright (2013)	Excision Arthroplasty	4.1	SVO: excellent (52.4%), good (14.3%), fair (9.5%), poor (23.8%); PFRA: excellent (31.6%), good (36.8%), fair (5.3%), poor (26.3%); PFIA: excellent (44.4%), good (33.3%), fair (0.0%), poor (22.2%); no statistical significance between groups	N/A	All caretakers were happy with the final results and would recommend the final surgery performed to others

Appendix 1 Figure 1. Surgical failures and hardware associated complications reported at the time of follow-up across different salvage procedures (arthrodesis, arthroplasty, excision), with complication categories displayed by frequency

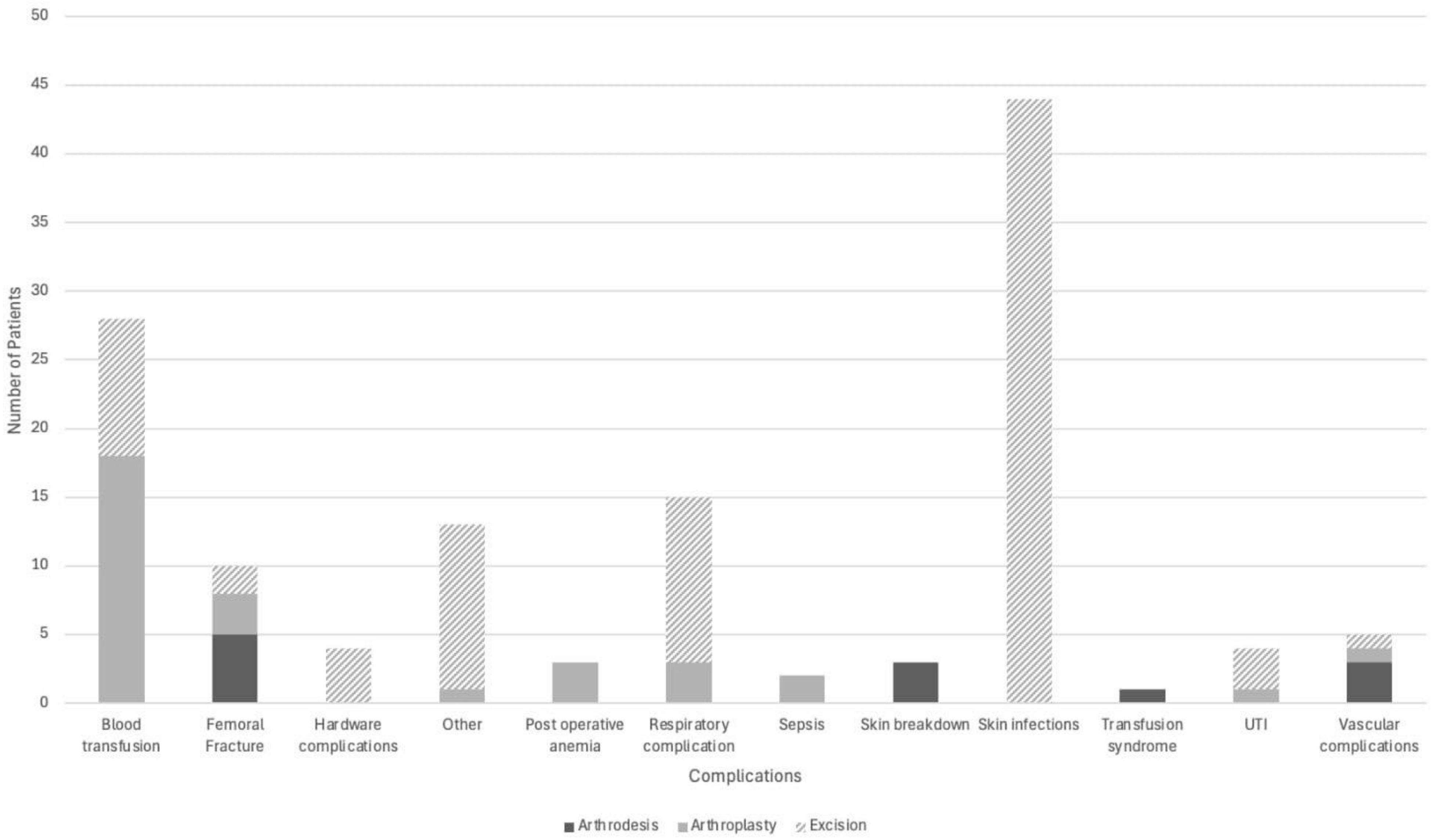

Number of Patients
50
45
40
35
30
25
20
15
10
5
0
Blood transfusion
Femoral Fracture
Hardware complications
Other
Post operative anemia
Respiratory complication
Sepsis
Skin breakdown
Skin infections
Transfusion syndrome
UTI
Vascular complications
Complications
Arthrodesis
Arthroplasty
Excision
